# Recruitment of complete crAss-like phage genomes reveals their presence in chicken viromes, few human-specific phages, and lack of universal detection

**DOI:** 10.1093/ismejo/wrae192

**Published:** 2024-10-03

**Authors:** María Dolores Ramos-Barbero, Clara Gómez-Gómez, Gloria Vique, Laura Sala-Comorera, Lorena Rodríguez-Rubio, Maite Muniesa

**Affiliations:** Departament de Genètica, Microbiologia i Estadística, Facultat de Biologia, Universitat de Barcelona, Avinguda Diagonal, 643, Prevosti Building, Floor 0. Barcelona E-08028, Spain; Departament de Genètica, Microbiologia i Estadística, Facultat de Biologia, Universitat de Barcelona, Avinguda Diagonal, 643, Prevosti Building, Floor 0. Barcelona E-08028, Spain; Departament de Genètica, Microbiologia i Estadística, Facultat de Biologia, Universitat de Barcelona, Avinguda Diagonal, 643, Prevosti Building, Floor 0. Barcelona E-08028, Spain; Departament de Genètica, Microbiologia i Estadística, Facultat de Biologia, Universitat de Barcelona, Avinguda Diagonal, 643, Prevosti Building, Floor 0. Barcelona E-08028, Spain; Departament de Genètica, Microbiologia i Estadística, Facultat de Biologia, Universitat de Barcelona, Avinguda Diagonal, 643, Prevosti Building, Floor 0. Barcelona E-08028, Spain; Departament de Genètica, Microbiologia i Estadística, Facultat de Biologia, Universitat de Barcelona, Avinguda Diagonal, 643, Prevosti Building, Floor 0. Barcelona E-08028, Spain

**Keywords:** Crassvirales, crAss-like phage, bacteriophage, Bacteroides, Gut viromes, fecal microbiota, biogeography

## Abstract

The order Crassvirales, which includes the prototypical crAssphage (p-crAssphage), is predominantly associated with humans, rendering it the most abundant and widely distributed group of DNA phages in the human gut. The reported human specificity and wide global distribution of p-crAssphage makes it a promising human fecal marker. However, the specificity for the human gut as well as the geographical distribution around the globe of other members of the order Crassvirales remains unknown. To determine this, a recruitment analysis using 91 complete, non-redundant genomes of crAss-like phages in human and animal viromes revealed that only 13 crAss-like phages among the 91 phages analyzed were highly specific to humans, and p-crAssphage was not in this group. Investigations to elucidate whether any characteristic of the phages was responsible for their prevalence in humans showed that the 13 human crAss-like phages do not share a core genome. Phylogenomic analysis placed them in three independent families, indicating that within the Crassvirales group, human specificity is likely not a feature of a common ancestor but rather was introduced on separate/independent occasions in their evolutionary history. The 13 human crAss-like phages showed variable geographical distribution across human metagenomes worldwide, with some being more prevalent in certain countries than in others, but none being universally identified. The varied geographical distribution and the absence of a phylogenetic relationship among the human crAss-like phages are attributed to the emergence and dissemination of their bacterial host, the symbiotic human strains of *Bacteroides*, across various human populations occupying diverse ecological niches worldwide.

## Introduction

The crAssphage (cross-assembly phage) group of phages first came to the attention of the scientific community in 2014 after its *in silico* identification in a metagenomic analysis of human feces [1]. Initially considered as a single entity, the first prototypical crAssphage (or p-crAssphage) was later described as belonging to the new order Crassvirales, family *Intestiviridae*, species *Carjivirus communis*. The order Crassvirales currently comprises four families, 11 subfamilies, 42 genera, and 73 new species (https://ictv.global/taxonomy) [1–5]. All members of Crassvirales, also known as crAss-like phages, exhibit similar genomic architecture, featuring a relatively large double-stranded circular DNA genome of ~100 kilobases. Crassvirales is a monophyletic group with a common evolutionary origin. Some of the phage proteins in this group are universally conserved across crAss-like phage subfamilies and share common ancestors [4, 5]. However, within the order, different branches can also be observed, and the taxonomy is continuously evolving with the discovery of new crAss-like phage members [6].

Although most crAss-like phages remain uncultured, analysis of the few isolated virions and the available genomes of crAss-like phages suggests they share a podovirus-like morphology and function as virulent phages, targeting bacteria within the phylum Bacteroidota, specifically the genus *Bacteroides* [4, 6].

Efforts to isolate crAss-like phages were unsuccessful until four years after the discovery of p-crAssphage, when ΦCrAss001 was isolated in *Bacteroides intestinalis* [7] and assigned to the family *Steigviridae*, species *Kehishuvirus primarius* [8]. The isolation of crAss-like phages in pure culture remains challenging, with only a few other crAss-like phages isolated to date: DAC15 and DAC17 (family *Steigviridae*, species *Wulfhauvirus bangladeshii*) [8], infecting *Bacteroides tethaiotaomicron* [9] in 2020; ΦCrAss002 (family *Intestiviridae*, species *Jahgtovirus secundus)* [8] infecting *Bacteroides xylanisolvens* in 2021 [10]; three new species in 2023 (family *Steigviridae*, species *Kehishuvirus tikkala* (Bc01), and *Kolpuevirus frurule* (Bc03), and family *Intestiviridae*, species *Rudgehvirus jaberico* (Bc11)) infecting *Bacteroides cellulosilyticus* [11]; although initially it was not classified, a *Cellullophaga* phage ɸ14:2, a marine isolate [12] was later included as a member of the Crassvirales order (family *Steigviridae*, species *Akihdevirus balticus*) [6]; and recently 25 new phages (crAssBcn phages 1–25) of the genus *Kehishuvirus*, belonging to at least six different species, infecting *B. intestinalis* [5]. All other crAss-like phages reported so far correspond to genomes generated as a result of composite assemblies and have never been propagated in pure culture in a laboratory, which limits their study.

p-crAssphage has been found to exhibit a high level of host specificity and high abundance in sewage and biosolids in Europe and the United States. Initially, it was thought to be less abundant in sewage samples from Asia and Africa [13, 14], but subsequent research using DNA sequences of p-crAssphage demonstrated its global prevalence [15]. Although crAss-like phages are thought to exhibit high human specificity, qPCR analysis has also revealed the presence of p-crAssphage in samples containing only animal feces [14, 16, 17], albeit at lower levels than in human feces [17]. In addition to p-crAssphage, previous taxonomical analysis described non-human, environmental phages in the Epsilon and Zeta groups of Crassvirales [6]. However, to date no more studies have assessed the level of human specificity of other members of the order Crassvirales and little information is available about the worldwide distribution of other crAss-like phages [4, 5].

To fully evaluate the Crassvirales group and to determine how this group of phages has spread across the human populations around the globe, a comprehensive understanding of their specificity and prevalence in the human gut is required. However, identifying human-specific crAss-like viruses within the vast diversity of Crassvirales remains an ongoing task and warrants further investigation.

In this study we used a custom database of 91 crAss-like phages. The database included only complete phage genomes to evaluate their abundance and prevalence in a collection of 1337 viral metagenomes with different levels of human pollution, from human feces, environmental samples (wastewater and soil), and food, as well as their absence in a collection of animals fecal viromes (feces of chickens, rabbits, cows, pigs, and non-human primates). The phylogenomic relationship and the geographical distribution of the human crAss-like phages were studied across metagenomes from a wide range of countries. This comprehensive metagenomic recruitment analysis aimed to determine which members of the order Crassvirales are exclusively found in humans, to ascertain whether the human-specific phages share common genomic traits and are phylogenetically related, and finally to delineate their biogeographical prevalence.

## Materials and methods

### Samples

This study evaluated the presence of 91 complete, non-redundant genomes of crAss-like phages selected from public databases (Supplementary Data 1) in 84 viromes obtained from human, food, environmental, and animal samples (Supplementary Data 2). Among these, environmental viromes including soil and wastewater, and viromes of dairy products (cheese, milk, and kefir), were obtained in this study. Viromes from human feces and urine, food viromes (vegetables, meat, and fish), and two viromes of chicken feces, were all generated by our group in previous studies [18–21]. A brief summary of the preparation methods for those viromes appears below. Other animal viromes (pig, rabbit, cattle, and non-human primates were generated by other authors and are available in the databases (Supplementary Data 2).

### Human samples

Seven viromes from human feces and 16 viromes from human urine were obtained as described in previous studies [19, 20]. All samples were collected from healthy adults, were anonymized, and all individuals were volunteers. The original study that generated the sequences used here was approved by the Clinical Ethics Committee of the Hospital de la Santa Creu i Sant Pau (12/065/1350) [20].

### Environmental samples

Six raw sewage samples were collected over one year period from the influent of two urban wastewater treatment facilities (Besòs and Prat) in Catalonia, northeastern Spain. Each facility serves a population of over 2 000 000 inhabitants. Samples were collected in sterile containers, transported to the laboratory within 2 h of collection, and processed immediately for the analysis of fecal indicators and bacteriophage isolation, as described below. Six wastewater viromes, three from each wastewater treatment plant were generated with these samples.

Two soil viromes were generated in this study from two pools of soil samples, each consisting of a mixture of five samples (5 g of each sample) from croplands in Barcelona (northeast Spain) and five samples from Asturias (north Spain). For each pool, 25 g was homogenized in 50 ml of PBS using stomacher bags with filters (Afora) in a Masticator (IUL Instruments GmbH) for 2 min. After the homogenization with 50 ml of PBS, phage particles were recovered by the addition of 1 g of beef extract (Sigma Aldrich) to the homogenate and increasing the homogenate pH to 8.5–8.7 with NaOH 0.1 N to detach viruses that can be adhered to soil particles (method adapted from [22]). The homogenate obtained was processed for purification of phage particles as described below.

### Food samples

Twenty-nine food virome samples were analyzed, four were generated in this study and the rest were obtained in a previous study from our group [18]. The meat pool included six viromes of chicken meat, two of beef, and two of pork. In the fish category, one pool was obtained from mussels, one of clams (shellfish 2), fresh hake (Atlantic fish 1), sardine (Atlantic fish 2), frozen hake (frozen fish 1), frozen monkfish (frozen fish 2), whiting (Mediterranean fish), salmon (farm fish 1), and trout (farm fish 2). The vegetable pool was comprised of two viromes obtained from lettuce, two from spinach, two from cucumber.

New viromes were generated in the category of vegetables, a nut milk product (horchata). In the category of dairy products, one virome of fresh milk, one of fresh cheese, and one of kefir Twenty-five g of kefir and fresh cheese, and 20 ml of fresh milk and unpasteurized horchata (nut-based milk) were homogenized/mixed for 2 min in 60 ml of phage buffer (22 mM KH_2_PO_4_, 50 mM Na_2_HPO_4_, 85 mM NaCl, 1 mM MgSO_4_, and 0.1 mM CaCl_2_) using the stomacher bags with filters (Afora) in a Masticator (IUL Instruments GmbH). The homogenates were centrifuged at 4000 *× g* and filtered through 0.22 μm polyethersulfone (PES) membranes. All samples were purchased from local retailers in Barcelona (Spain) in 2017–2020, transported in sterile containers, and kept at −80°C until analysis.

### Non-human samples

Twenty-four viromes of animal feces were selected from public databases, including four chicken fecal viromes from Spain and China, two additional viromes of chicken feces, eight viromes of pig feces, one virome of cattle feces, two viromes of rabbit feces, three non-human primate viromes, and four viromes of makaka feces.

### Culturable bacterial and viral fecal indicators

Samples were evaluated for the presence of fecal indicators, either in the present (environmental samples, horchata, and dairy) or in a previous study from our group [18]. Samples from animal feces were not processed in this study as the data for these viromes were obtained from public databases. *Escherichia coli* in samples or homogenates was determined on Chromocult Coliform Agar (Merck). Initial incubation was performed for 2 h at 37°C to acclimatize potentially damaged microorganisms and then overnight at 44°C. Ten per cent of the colonies turned blue, representing potential *E. coli*, and were confirmed with the Indole test and grown on McConkey agar. Somatic coliphages, proposed as viral indicators of fecal pollution [23], were analyzed after homogenate filtration to evaluate the presence of infectious fecal viruses in the samples. The filtrates were decimal diluted in PBS and somatic coliphages in one ml of each dilution were analyzed by the double agar layer method following the ISO standard [24], using *E. coli* strain WG5 (ATCC 700078) as host, and incubation at 37°C for 18 h. Each dilution was analyzed in triplicate. Each homogenate was analyzed in duplicate. Mann–Whitney U-test was performed to assess the statistically significant differences in *E. coli* or in somatic coliphages concentrations between the group of samples with high (human gut, human urine, wastewater 1 and 2) or low fecal load (soil, meat, fish, vegetable, dairy). For statistics, GraphPad Prism (GraphPad Software) was used. A difference was deemed significant when the *P*-value ≤ 0.05.

### Purification of phage particles

For the viromes generated in this study, 50 ml of wastewater samples and 50 ml of the homogenate of soil samples, nut milk, and dairy products were filtered through 0.22 μm pore size, low-protein-binding PES membranes (Millipore) to remove bacteria and other particulate material. Filtered homogenates were 20-fold concentrated using polyethylene glycol (PEG) precipitation [25], followed by incubation at 4°C for 12 h, and centrifugation at 16000 *× g*. Two ml of the PEG-concentrated suspensions were dialyzed and treated twice with chloroform 1:10 (*v:v*), mixed by vigorous vortexing for 5 min, and centrifuged at 16000 *× g* for 10 min. Chloroform treatment aims to break bacterial cells and any DNA-containing small vesicles that might have passed through the filters. The supernatant was treated with DNase I (100 units/ml; Sigma-Aldrich) for 1 h at 37°C to eliminate any non-packaged DNA. DNase was inactivated by heating for five min at 75°C. Human samples [19, 20], chicken feces [21], and the rest of food viromes [18]were processed in previous studies using the same protocol described above with minor modifications.

### Phage DNA isolation

DNA isolation from the purified particles was performed with QIAamp DNA Blood (Qiagen GmbH), following the manufacturer’s instructions, and the obtained DNA was suspended in 200 μl of sterile bi-distilled water. The DNA concentration of each pooled sample was evaluated using a Qubit Fluorometer (Life Technologies), and the DNA quality was further confirmed by the 2100 Bioanalyzer system (Agilent Technologies).

### Sequencing

Five μl of DNA at a concentration of 0.2 ng/μl was fragmented and used to prepare libraries for whole genome sequencing using the Nextera XT Kit (Illumina) according to the manufacturer’s protocol. The libraries were purified with AmPure beads (Beckman Coulter Inc.), checked for fragment distribution and size, and quantified using a TapeStation 4200 and the Agilent High Sensitivity D1000 ScreenTape system (Agilent Technologies) in a Quantus Fluorometer (Promega). An equimolar pool of the 25 individual phage genomes was separately sequenced on the NextSeq System (Illumina) with a high output run of 300 cycles.

### Metagenomic sequence trimming

Metagenomic raw reads generated in this study and those downloaded from public databases were trimmed separately using Trimmomatic (LEADING:3 TRAILING:3 SLIDINGWINDOW:4:15 MINLEN:36) [26]. Finally, trimmed metagenomes were pooled by origin (human gut, wastewater, food) as detailed in Supplementary Data 2. The quality of trimmed reads was checked by FastQC [27].

### Presence, abundance and diversity of crAss-like phages in different viromes

To assess the presence of crAss-like phages in the different viromes, reads from the viromes in the database, which includes human, animal, and low fecal contamination viromes from this study (Supplementary Data 2), were recruited against the genomes of crAss-like phages. Selected crAss-like phage genomes have been used as subject in the BLASTn database and virome reads were the queries [28]. The presence and relative abundance of the human crAss-like phages were determined using stand-alone BLASTn with a cutoff of 70% query coverage, an *e*-value ≥0.1, and filtered by the “best hit” option by BlastTab.best_hit_sorted.pl script from the enveomics collection [29]. The default BLASTn parameters of VIRIDIC were used (−word_size 7 -reward 2 -penalty −3 -gapopen 5 -gapextend 2'), specie threshold 95%, and genus 70% of similarity) [28]. After applied the best-hit and query coverage cutoff, the relative abundances of each crAss-like phage in each virome were calculated using BlastTab.seqdepth.pl from enveomics collection [29]. Sequencing depth (SD) values were normalized by dataset size (Gbp) and genome length (Kbp) (SD/Gbp/Kbp). Fragment recruitment data were plotted using the R package from the enveomics collection [29]. The graphs (bars, boxplots, and heatmaps) were drawn using Plotly in R [30]. Finally, the average nucleotide identity of mapped reads (ANIr) of each virome was determined as previously described [31]. Only reads with ≥70% query coverage were considered for the analyses. ANIr and relative abundance heatmaps were drawn by heatmapper [32].

### Genome comparison and phylogenetic analysis of human-specific crAss-like phages

Phage genomes were clustered at 100% identity by cd-hit-est [33]. Cd-hit-est does not cluster together genomes that are 100% identical but circularly permuted with different start sites. Therefore, VIRIDIC (BLASTn-based) [28], which checks each comparison for genomic synteny and calculates the intergenomic similarity of two viruses with BLASTn [34], was also used to assess similarities between phage genomes. Whole genome alignments were performed by Mauve of Geneious Prime version 2024.0.5 [35]. ViPTree v 4.0 [34] was used to create a viral "proteomic tree" with the phage genome sequences available in the published databases. The tree is based on a distance matrix of pairwise genome-wide similarities calculated using tBLASTx. The proteomic tree illustrates the global genomic similarity relationships among the selected phages and facilitates alignment visualization for comparative genomics of viruses.

### Presence and abundance of human crAss-like phages worldwide

To evaluate the geographical distribution of the highest human-specific crAss-like phages, a collection of viral metagenomes was created in this study (updated from a previously reported global human viromes database) [5]. This collection comprised 1260 human gut viromes available in the databases from children and adults across 14 countries (Table 1; see Supplementary Data 3 for more extended information) that were recruited against the 13 human phages. The presence and relative abundance of the human crAss-like phages were determined as described above. Next, crAss-like phage abundances were calculated using sequencing depth (SD) as previously described, values normalized by dataset size (Gbp) and genome length (Kbp) (SD/Gbp/Kbp). Fragment recruitment data were plotted by the enveomics R package in the R statistical tool [29]. The graphs (bars, boxplots, and heatmaps) were drawn with Plotly by R [30].

**Table 1:** **Origin and characteristics of the metaviromes used in this study**. Metagenomes in bold correspond to those that recruited against specific crAss-like phages.

Country	# metaG_w notes	# of people	Age	Health state	Clean read Bps by country/sequencing platform	VLP or Bulk	MDA	Sample type	Extraction method	Detected crAss-like phages	Ref.
**Ethiopia**	**29**	**269**	**Children**	**Diarrheal disease**	**4 225 680 651**	**VLP**	**Yes**	**Feces**	**C, F, N**	**Yes**	[36]
**USA**	**16**	**10**	**Adults, Teens**	**Fecal transplantation**	**9 373 845 702**	**VLP**	**Yes**	**Feces**	**C, F, CE, N**	**Yes**	[37]
**USA**	**24**	**24**	**Children**	**IBD, Healthy**	**2 577 552 227**	**VLP**	**Yes**	**Feces**	**C, F, N**	**Yes**	[38]
**China**	**196**	**196**	**Adults**	**Hypertension, Healthy**	**1.05501 x10** ^**12**^	**Bulk**	**No**	**Feces**	**N/A**	**Yes**	[39]
**Finland**	**96**	**38**	**Infants**	**Type 1 diabetes, Healthy**	**15 942 577 574**	**VLP**	**Yes**	**Feces**	**C, F, N**	**Yes**	[40]
**Ireland**	**20**	**20**	**Infants**	**Various birth modes**	**5 488 316 876**	**VLP**	**Yes**	**Feces**	**C, F, P, N**	**Yes**	[41]
**Uganda**	**65**	**65**	**Adults**	**HIV, Healthy**	**32 402 875 119**	**VLP**	**Yes**	**Feces**	**C, F, N**	**Yes**	[42]
Spain	19	19	Adults	IBD, Healthy	6 246 589	VLP	Yes	Feces	C, F, P, N	Bdl	[43]
**Tanzania**	**27**	**27**	**Adults**	**Healthy**	**60 267 567 734**	**Bulk**	**No**	**Feces**	**N/A**	**Yes**	[44]
Peru	36	36	Adults	Healthy	1.23152 x10^11^	Bulk	No	Feces	N/A	Bdl	[44]
Italy	11	11	Adults	Healthy	13 446 170 791	Bulk	No	Feces	N/A	Bdl	[44]
USA	22	22	Adults	Healthy	96 979 018 221	Bulk	No	Feces	N/A	Bdl	[44]
**Malawi**	**320**	**53**	**Children, Adults**	**Malnutrition, Healthy**	**2 387 517 871**	**VLP**	**Yes**	**Feces**	**C, F, N**	**Yes**	[45]
**Ireland**	**84**	**13**	**Adults**	**IBD, Healthy**	**1.31496 x10** ^**11**^	**VLP**	**Yes**	**Feces**	**C, F, P, N**	**Yes**	[46]
**Cameroon**	**63**	**221**	**Infants, Adults**	**Healthy (contact with bats)**	**73 694 302 905**	**VLP**	**Yes**	**Feces**	**C, F, N**	**Yes**	[47]
Estonia	51	4	Infant	Type 1 diabetes, Healthy	19 806 647 068	VLP	Yes	Feces	C, F, N	Bdl	[48]
Finland	169	18	Infant	Type 1 diabetes, Healthy	64 749 010 013	VLP	Yes	Feces	C, F, N	Bdl	[48]
**Spain**	**7**	**7**	**Adults**	**Antibiotic, Healthy**	**75 043 928**	**VLP**	**No**	**Feces**	**C, F**	**Yes**	[20]
**Mexico**	**5**	**28**	**Children**	**Obesity**	**885 441 818**	**VLP**	**No**	**Feces**	**C, F**	**Yes**	[49]

MDA: Multiple displacement amplification. VLP: Virus like particles. C: centrifugation. F: filtration. N: nucleases. CE: Centricon concentration. P: PEG precipitation. IBD: Inflammatory bowel disease

## Results

A crAss-like phage database was constructed in this study using 91 non-redundant complete phage genomes identified as crAssphage or crAss-like phage available in public databases (Supplementary Data 1). Of these, 25 crAssBcn phages were recently isolated in our area (Barcelona) [5]. The only isolated virions represented in this list of genomes were ΦCrAss002, from the *Intestiviridae* family, ΦCrAss001, DAC15, DAC17, and the 25 crAssBcn phages (from the *Steigviridae* family) [5, 7, 9, 10], whereas the rest (Supplementary Data 1) were uncultured viruses.

### Presence and relative abundance of crAss-like phage genomes in human viromes and viromes containing human fecal pollution

**Figure 1:**
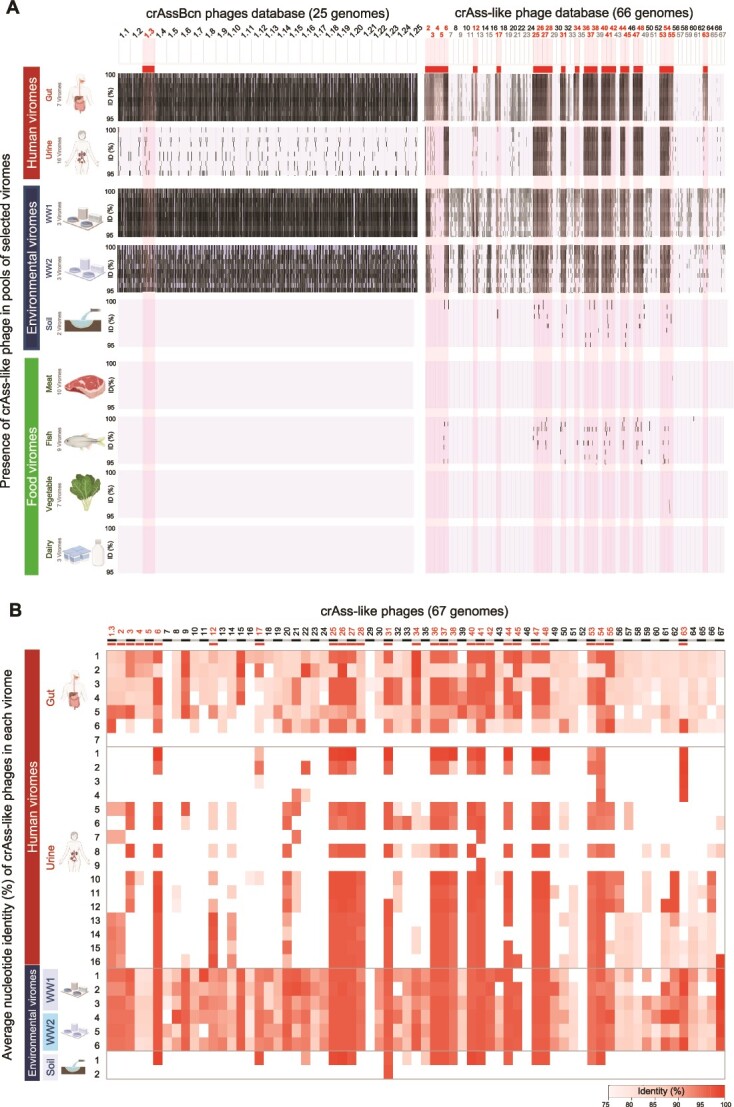
**Presence of crAss-like phages in human-impacted viromes.** (**A**) Presence of CrAss-like phage in the selected viromes. Reads from various viromes from humans (feces and urine), environmental (urban wastewater from plant 1 and 2(WW) and agricultural soil), meat pool (chicken, pig, and beef meat), fish pool (Atlantic, Mediterranean, frozen, farm fish, and shellfish), vegetables pool (spinach, lettuce, cucumber, and horchata) and dairy products pool (milk, cheese, and kefir), recruited against the genome of 25 crAssBcn phages and 66 crAss-like phages from databases (x-axis). Selected phages are indicated in the top of each chart. Recruitment plots for each virome display mapped reads and the percent identity of the reads for each phage genome are shown on the y-axis (only reads with 95 to 100% identity are shown). (**B**) Heatmap of identity of detected crAss-like phages in viromes. Average nucleotide identity of mapped reads (ANIr) from each virome was calculated for each crAss-like genome separately (only reads recruited over 70% of coverage were considered). Viromes with crAss-like phage relative abundances below 0.00001 SD/Kbp/Gbp were not showed. (see Supplementary Data 2 for numerical data used to build the Heatmap).

To ascertain which members of the order Crassvirales were present in humans, the 91 complete genomes were recruited in a set of 60 viromes of human origin or impacted by humans (Supplementary Data 2), comprising 23 human samples, eight environmental samples, and 29 food samples. In the recruitment plots (Fig. 1A), the reads of each virome are mapped against each complete phage genome to verify phage presence/absence in the virome. An intense vertical black signal indicated high recruitment with a read coverage of over 70% (only reads that mapped with 95–100% identity are shown in the recruitment plot) and allowed selection of phage genomes showing high recruitment (Fig. 1). Weak signals indicated that reads only recruited against part of the phage genome and not to the whole phage genome, in which case these phages were considered not to be present in the virome. As the recruitment results showed a similar behavior for all crAssBcn phages (Fig. 1A), only one representative of the group (the genome of phage ɸCrAssBcn3) was used in subsequent analyses, as shown below. Read recruitment against phages was observed in the pool of human feces (comprising seven gut viromes) [19, 20], human urine (comprising 16 urine viromes) [19, 20], and in wastewater viromes, which were distributed in two pools, one containing three viromes from wastewater treatment plant 1 (Besòs) and one containing three viromes from wastewater treatment plant 2 (Prat), both obtained in the present study (Fig. 1A). Heatmap shows the average nucleotide identity of mapped reads (ANIr) from each individual virome for each crAss-like genome separately and revealed high ANIr in soil virome 1 for several crAss-like phages (Fig. 1B, Supplementary Data 2 for numerical data).

The fecal content of all samples used to obtain the viromes was evaluated. Human samples (feces and urine) and urban wastewater showed the highest levels of the bacterial fecal indicator (*E. coli*) and viral fecal indicators (somatic coliphages), reflecting their high fecal load (Supplementary Fig. 1). Differences in fecal indicators between highly polluted samples and low polluted samples (soil and food) were significant by the Mann–Whitney U-test (*E. coli*, *U* = 20.0, *P* < 0.0159 and somatic coliphages *U* = 20.0, *P* < 0.0159). As an effective indicator should also be detectable in samples expected to have a low fecal load, viromes from agricultural soil samples (obtained in this study) and food (vegetables, meat, fish, and dairy products) [50] (Supplementary Data 2), which had significantly less fecal contamination than the human samples (Supplementary Fig. 1), were used to recruit crAss-like phage genomes.

Many but not all of the complete crAss-like phage genomes were detected in samples with high levels of human fecal contamination (Fig. 1). Recruitment against crAss-like phages was higher in human feces and wastewater viromes, and lower in human urine viromes. In the case of crAssBcn phages, they were highly recruited in human feces and wastewater viromes but were less represented in urine viromes compared to other crAss-like phages.

In food samples and agricultural soil, the amount of human fecal pollution was very low (Supplementary Fig. 1). Regardless of the origin of the fecal pollution, crAss-like phages were not detected in these viromes (below recruitment detection limits), or only a few reads were recruited in one of the soil viromes against some of the phages (Fig. 1B).

The relative abundance of each crAss-like phage in each pool (Supplementary Fig. 2) is in accordance with the recruitment plots (Fig. 1A) and the ANIr (Fig. 1B). The highest relative abundance of phages corresponded to the human gut, urine, wastewater and soil viromes, being lower in food viromes. The relative abundance of crAssBcn phages was higher compared to the other crAss-like phages analyzed. In accordance with the recruitment plots (Fig. 1), crAss-like phages were not observed in food viromes that had very low levels of human fecal pollution.

### Selecting crAss-like phages with the highest specificity to human viromes or viromes containing human fecal pollution

Using the selection criterion of “presence in human viromes”, crAss-like phages with a horizontal genome coverage (defined as the portion of the phage genome covered by metagenomic reads) of over 40% in the human gut and human urine were chosen (Supplementary Data 4). These corresponded to 27 crAss-like phages and all the crAssBcn phages (Supplementary Data 1). As indicated above, ɸCrAssBcn3, one of the crAssBcn phages with a high percentage of recovery (Supplementary Data 1), was used to represent the whole group. In total, the 28 crAss-like phages with the best recruitment results in human viromes were included in the study.

The 28 crAss-like phages selected (Supplementary Data 1 and Fig. 1) were also detected in wastewater viromes. In this case, coverage levels were close to 40% in only 19 phages, 20–30% in four phages, and below 10% in the remaining five phages. Sequence diversity was lower in fecal than in wastewater samples, which are inherently more complex matrices, as discussed below. The higher diversity of wastewater viromes reduces the likelihood of detecting a particular genome in a sample, potentially contributing to a lower recruitment in wastewater viromes, even if phages are abundant in human feces viromes. Therefore, all phages present in human fecal viromes were selected for the following analysis regardless of their relative abundance in wastewater viromes.

### Absence of selected crAss-like phages in animal gut viromes

**Figure 2:**
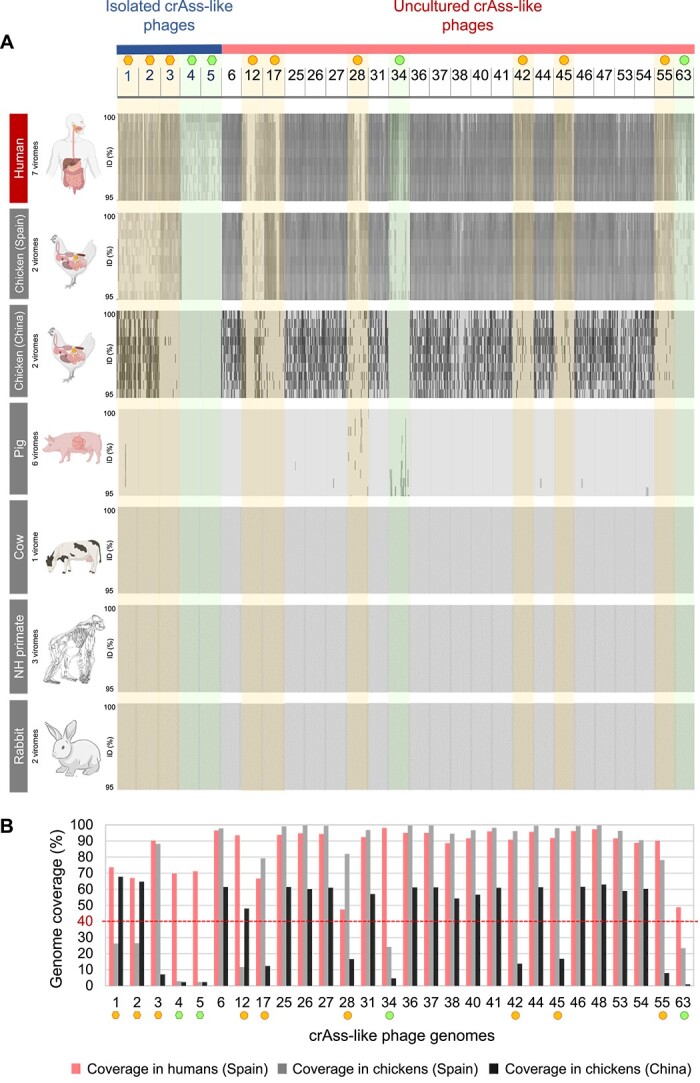
**Recruitment plots of crAss-like phages detected in human-impacted viromes with reads in animal viromes.** The datasets displayed were obtained from healthy human feces, chicken feces (from Spain and China), pig feces, cow feces, non-human primates (makaka) intestinal content, and rabbit feces (for more information see Supplementary Data 2). (**A**) the recruitment plots display the results of a BLASTn search of metagenomic short reads from animal viromes against the complete genomes of 28 selected crAss-like phages identified in human viromes (X-axis). The upper panel shows the number given to each crAss-like phage genome according to its position in the list of phages used in the study (Supplementary Data 1). Only viromes showing recruitments are included. The recruitment plots for each metagenome display mapped reads and the percent identity of the reads for each phage genome (y-axis) (only reads with 95 to 100% identity). (**B**) the chart indicates the percentage of genome coverage of the 28 selected human phages in viromes of humans, chickens from Spain, and chickens from China. Those with coverage of more than 40% in the human gut and less than 40% in both chicken viromes were selected as highly human phages (green). Those with coverage of more than 40% in the human gut and less than 40% in at least one of the two chicken viromes were considered as quite good human phages (orange). Uncultured phages are marked by a circle and isolated phages are indicated by a hexagonal symbol.

The 28 crAss-like phages selected for their abundance in human feces were analyzed in a collection of 24 animal fecal viromes of chicken, pigs, cows, rabbits, and non-human primates (Supplementary Data 2). As previously, to objectively apply the exclusion criteria (“not detected in animal feces”), we selected the phages with a horizontal genome coverage of below 40% in the animal viromes. No reads of cows, rabbits, and non-human primate viromes were recruited against this set of phages, and only anecdotally in pig viromes. In contrast, consistently high recruitment against diverse crAss-like phages was obtained with two different sets of chicken viromes, one from Spain and the other from China (Fig. 2). Further analysis revealed that 15 crAss-like phages were present in both chicken viromes with more than 40% of genome coverage (Fig. 2B), and these were consequently excluded due to their lack of human-specificity. In contrast, nine crAss-like phages were present in only one of these two chicken viromes (Fig. 2B, marked by orange symbols and labeled “quite” in Table 2). As human fecal contamination of the chicken samples could not be ruled out, and we did not want to exclude phages based on the results of viromes from a single geographical area, we decided to include selected crAss-like phages with less than 40% of genome coverage in at least one of the chicken viromes. Additionally, the four highly human-specific phages not detected (below 40% of genome coverage) in any animal viromes (Fig. 2B, marked by green symbols and labeled “high” in Table 2), were selected.

**Table 2:** **List of the 13 selected human phages**. Selection was based on their presence in samples with human fecal pollution and total or low recruitment in animal samples (coverage: > 40% in black and < 40% in red). In bold, the four phages showing the highest human specificity (High). WW: wastewater.

Phage #	Genome ID	Name		Family	Origin (City, Country)	Phage	Coverage (%)				Human specificity
		Common	Binomial				Human Gut	Urban WW	Poultry (Spain)	Poultry (China)	
1	OQ221538	ɸCrAssBcn3	*Kehishuvirus* sp*.*	*Steigviridae*	Barcelona, Spain	Isolated	74.0	69.0	26.2	67.8	Quite
2	NC_049977.1	ɸCrAss001	*Kehishuvirus primarius*	*Steigviridae*	Cork, Ireland	Isolated	67.3	67.1	26.4	64.7	Quite
3	MN917146.1	ɸCrAss002	*Jahgtovirus secundus*	*Intestiviridae*	Cork, Ireland	Isolated	90.3	9.8	88.2	7.0	Quite
**4**	**MT074136.1**	DAC15	*Wulfhauvirus bangladeshii*	*Steigviridae*	Stanford, USA	Isolated	**70.1**	**6.0**	**2.8**	**2.3**	**High**
**5**	**MT074138.1**	DAC17	*Wulfhauvirus bangladeshii*	*Steigviridae*	Stanford, USA	Isolated	**71.4**	**6.1**	**2.4**	**2.3**	**High**
12	MT774388.1	cr112	*Kehishuvirus splanchnicus*	*Steigviridae*	Cork, Ireland	Uncultured	49.1	51.2	23.4	48.1	Quite
17	MT774396.1	cr53	*Blohavirus americanus*	*Suoliviridae*	Cork, Ireland	Uncultured	66.7	27.8	79.2	12.3	Quite
28	MT774375.1	cr50	*Cohcovirus hiberniae*	*Suoliviridae*	Cork, Ireland	Uncultured	47.6	19.9	82.0	16.6	Quite
**34**	**MT774390.1**	cr85	*Kahnovirus oralis*	*Steigviridae*	Cork, Ireland	Uncultured	**98.1**	**38.9**	**24.1**	**4.6**	**High**
42	MT774401.1	cr6	*Afonbuvirus faecalis*	*Suoliviridae*	Cork, Ireland	Uncultured	90.9	24.4	96.2	13.7	Quite
45	MZ130492.1	cr12	*Afonbuvirus intestinihominis*	*Suoliviridae*	Cork, Ireland	Uncultured	91.9	25.6	98.0	16.8	Quite
55	MT774377.1	cr107	*Jahgtovirus intestinihominis*	*Intestiviridae*	Cork, Ireland	Uncultured	90.2	9.0	78.1	7.9	Quite
**63**	**MZ130488.1**	cr17	*Endlipuvirus intestinihominis*	*Intestiviridae*	Cork, Ireland	Uncultured	**93.5**	**7.0**	**11.6**	**0.9**	**High**

In total, a list of 13 human crAss-like phages was obtained, four of which stood out as the highest human-specific (Table 2): two (DAC15 and DAC17, both *Wulfhauvirus bangladeshii* family *Steigviridae*) were isolated phages, whereas cr85 (*Kahnovirus oralis*, family *Steigviridae*) and cr17 (*Endlipuvirus intestinihominis*, family *Intestiviridae*) corresponded to uncultured phages. Other isolated phages, namely ΦCrAss001, ΦCrAss002, and ɸCrAssBcn3 (Table 2), were categorized as quite specific for humans, given that ΦCrAss001 and ΦCrAss002 were identified in a Chinese chicken virome and ɸCrAssBcn3 in a Spanish chicken virome (Fig. 2). Of the 13 human phages, four showed high recruitment in wastewater (close to or higher than 40% coverage), phage cr85 being the most recruited in wastewater viromes among the four phages with the highest specificity (Table 2).

### Comparing the genomes of human-specific phages

**Figure 3:**
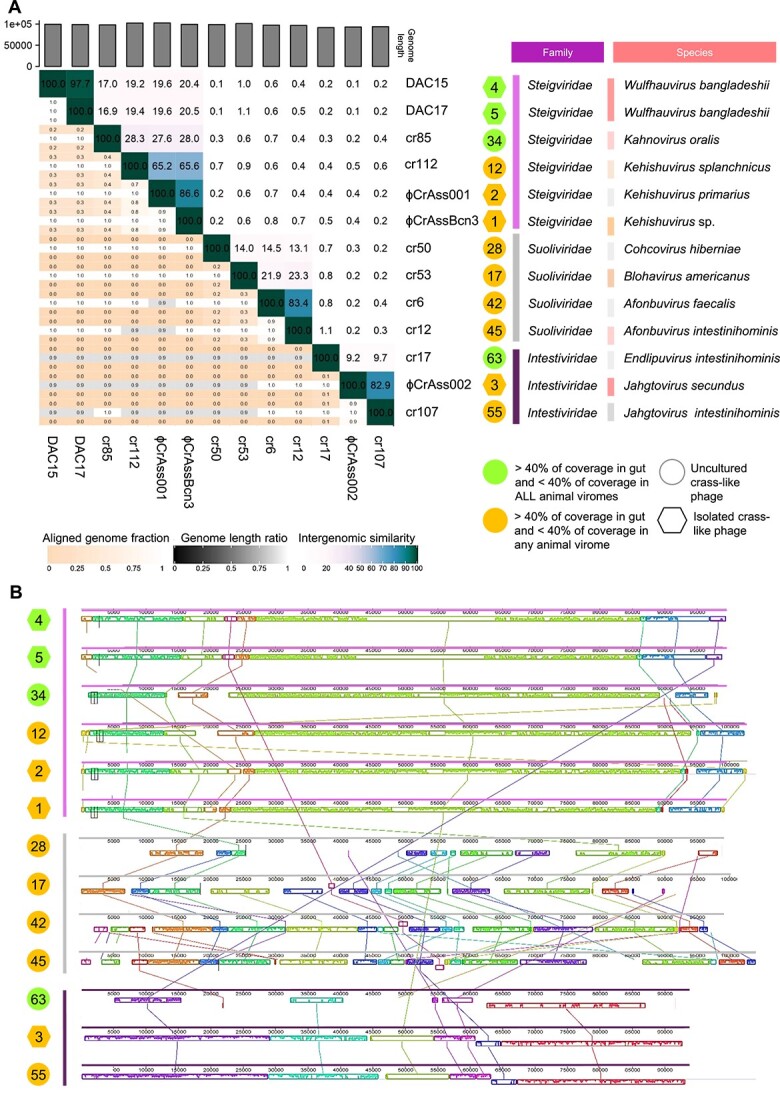
**Intergenomic comparison of the 13 human crAss-like phages genomes**. (**A**) the heatmap generated by VIRIDIC shows intergenomic similarity values (right half) and alignment indicators (left half). The percent identity between two genomes was determined by BLASTn, integrating intergenomic similarity values with data on genome lengths and aligned genome fractions. In the right half, darker colors signify closer genomic relationships. The numbers inside the map represent the identity values for each genome pair rounded to the first decimal. In the left half, aligned genome lengths are indicated, with darker colors corresponding to smaller aligned genome fractions. The family cluster and species cluster in which each phage is classified are indicated on the right side of the heatmap. (**B**) Multiple genome alignment constructed with the complete genomes of the 13 human-specific crAss-like phages. Boxes with identical colors indicate homologous DNA regions shared by two or more genomes. Uncultured phages are indicated by a circle and isolated phages by a hexagonal symbol. The number of each crAss-like phage indicates its position in the list of crAss-like phages used in the study (Supplementary Data 1).

The genomes of the 13 human- crAss-like phages were aligned to evaluate if they shared a core region. This finding would provide insights about common genomic features that could explain their specificity for humans. Moreover, the discovery of common nucleotide sequences in the human-specific phages would facilitate the development of molecular tools for their detection as human fecal markers. However, a genomic comparison by VIRIDIC analysis showed high sequence heterogeneity among the 13 human crAss-like phages. According to the International Committee for the Taxonomy of Viruses [51], the main species demarcation criterion for bacterial and archaeal viruses is currently set as a genome sequence identity of 95% over 85% of the complete genome. Applying this criterion, the 13 human crAss-like phage genomes were distributed in three families, eight genera, and 12 species (Fig. 3A), indicating that human-specificity is not restricted to a given family or species. No core genome region was observed when aligning the genomes of the 13 human crAss-like phages (Fig. 3B). High identity (over 80% in nucleotides), in accordance with their taxonomical classification, was found between phages CrAssBcn3 and CrAss001 (*Kehishuvirus* sp. and *Kehishuvirus primarius* respectively, family *Steigviridae*), DAC15 and DAC17 (both *Wulfhauvirus bangladeshii*, family *Intestiviridae)*, cr6 and cr12 (*Afonbuvirus faecalis* and *Afonbuvirus intestinihominis* respectively, both from family *Suoliviridae*, and CrAss002 and cr107 (*Jahgtovirus secundus* and *Jahgtovirus intestinihominis*, respectively, both from family *Intestiviridae*), but not between the rest. A lack of coincidence was also observed when comparing only the four phages highly specific for humans (Supplementary Fig. 3).

**Figure 4:**
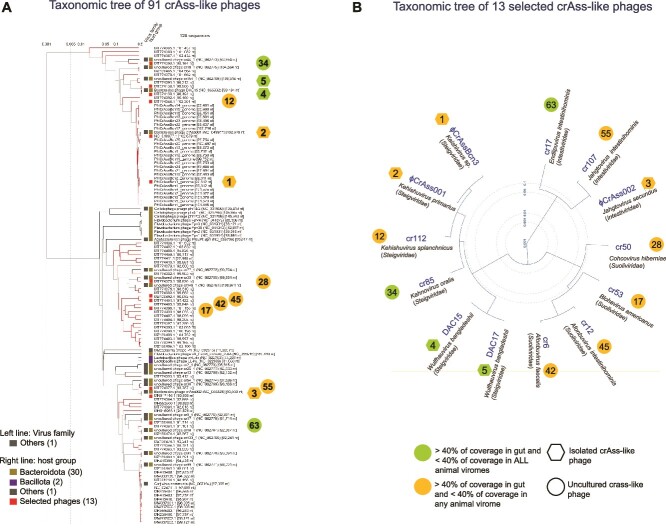
**Taxonomical analysis and phylogenomic relationships of the 13 human crAss-like genomes.** (**A**) Portion of a viral proteomic tree constructed with ViPTree, where the 13 phage genomes were included as a query and compared with crAss-like phage genomes available in the databases. Branch length is indicated at the top of the tree. (**B**) Phylogenetic tree constructed with the genomes of the 13 human crAss-like phages. Uncultured phages are indicated by a circle and isolated phages by a hexagonal symbol. The number of each crAss-like phage indicates its position in the list of crAss-like phages used in the study (Supplementary Data 1).

A phylogenomic comparison of all 91 crAss-like phage genomes included in the study revealed that the 13 human-specific crAss-like phages are distributed throughout the order Crassvirales (Fig. 4A). Three of the most human-specific ones are located closest to each other (all three belonging to the family *Steigviridae*), but cr17 (family *Intestiviridae*) is situated in a different branch of the tree. To better understand the phylogenomic relationships among the 13 human-specific phages, they were compared with each other and showed a distribution in three independent clusters that correspond to the three different families, *Steigviridae*, *Intestiviridae*, and *Suoliviridae*) (Fig. 4B).

The distribution detected in this analysis with the subset of 13 crAss-like phages is in accordance with the taxonomic classification. Those that belong to similar genera are closely related, whereas those of different families are only related at the order level, so no nucleotide similarities are expected.

### Biogeographical distribution of the human-specific crAss-like phages

**Figure 5:**
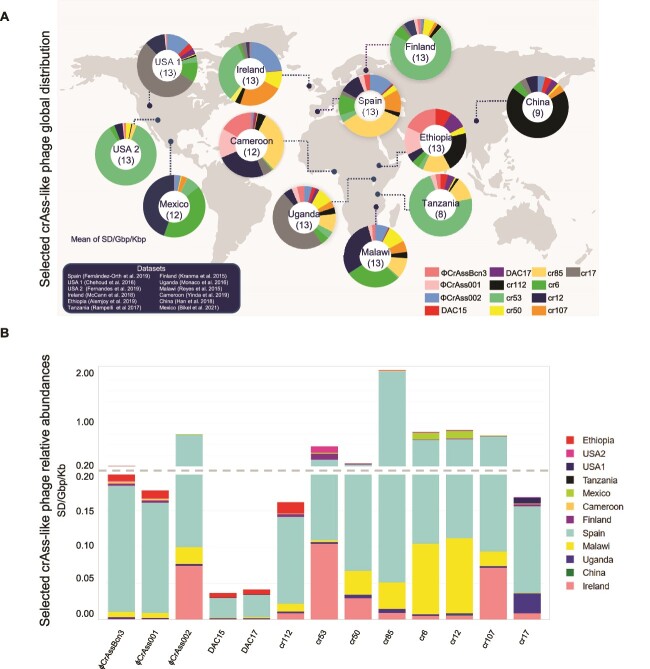
**Geographical distribution of the 13 human crAss-like phages**. (**A**) the distribution (percentage) of the 13 human crAss-like phages in each country. Only metagenomes showing recruitments are included, which are from Ethiopia, USA (USA 1 and 2), Tanzania, Mexico, Cameroon, Finland, Spain, Malawi, Ireland, Uganda, and China. The number of crAss-like phages found in each country is given in brackets. (**B**) Relative abundance of each of the 13 human phages in the different countries showing recruitments. Only mapped reads with coverage of at least 70% and best-hit reads were considered. Relative abundances of the human crAss-like phages were normalized by metagenome size (Gbp) and phage length (Kbp). World map figure in the background was obtained from Designspot/Freepik.

Our aim was to evaluate the geographical distribution of the 13 human crAss-like phages, and to assess whether some of them have a wider global distribution than others. This attribute was previously reported for p-crAssphage [15], using DNA genome fragments, and for crAssBcn phages [5]. Read recruitment of a viral metagenomes collection, consisting of 1260 human gut viromes from children and adults from 14 countries was conducted against the whole genomes of the 13 human crAss-like phages (Table 1; Supplementary Data 3). Among these, 77% (971) of the metagenomes, distributed in 11 countries, recruited against at least one of the 13 human phages (Fig. 5), whereas 22.9% (289 gut viromes) were below the detection limits.

The distribution of crAss-like phages varied between countries, with some showing more frequent recruitment and relative abundance than others (Fig. 5A). The country with the lowest diversity was Tanzania, with eight crAss-like phages, followed by China with nine. Mexico and Cameroon both had 12 phages, whereas the other countries were found to have all 13 crAss-like phages. No single crAss-like phage predominated in all the countries. A relatively high abundance was found for cr53 in Finland, Tanzania, USA 2, and Ireland, and for cr12 in Malawi and Mexico. Phage cr112 (*Kehishuvirus splanchnicus*) was predominant in China, and cr17 in USA 1 and Uganda. All these genomes are of uncultured phages. Regarding the isolated phages, ΦCrAss002 was the most predominant overall (the most prevalent in USA 1, Ireland, Spain, Uganda, and Malawi), whereas ΦCrAssBcn3 and ΦCrAss001 predominated in Ethiopia, Cameroon, Finland, and USA 2, and DAC15/DAC17 in China and Tanzania.

The percentage of crAss-like phages identified in viromes around the world (Fig. 5A) allows comparison between different countries. Although the metagenomic sequencing depth (SD) of all viromes was normalized by dataset size (Gbp) and genome length (Kbp), comparisons between countries had limitations. A phage may be very prevalent in one country compared to the other phages in the same country but could show a low global recruitment. To better evaluate phage prevalence globally, comparing the relative abundance of each phage across all countries is more informative (Fig. 5B). Hence, although phage cr112 predominated in China and Ethiopia, its global relative abundance was quite low. Similarly, DAC15 and DAC17 showed the lowest relative abundance overall, yet were identified in several countries. The highest recruitment of DAC15/DAC17 genomes was found in Spain, but they were not the most prevalent phages in this country. By far the highest relative abundance worldwide was shown by phage cr85 (Fig. 5B), followed by cr12, cr6, and ΦCrAss002. The most abundant isolated crAss-like phage was ΦCrAss002 (Fig. 5A).

**Figure 6:**
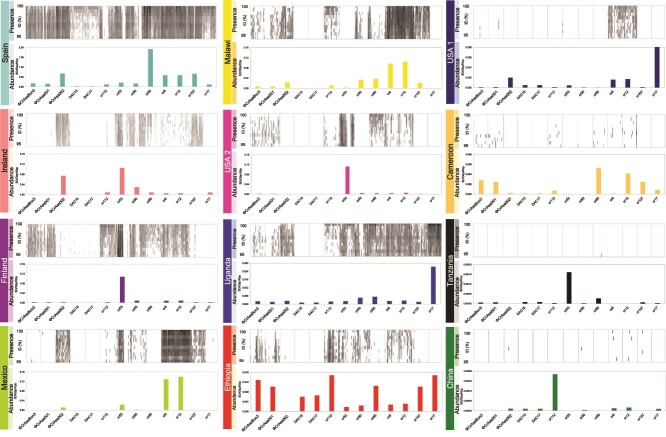
**Selected crAss-like phage presence (over 95% ID) and abundance around the globe.** Recruitment of the metagenomes from different countries against the 13 human crAss-like phages is displayed. For each country, the upper chart (presence) shows the recruitment plots with mapped reads (X-axis) and percent identity (95 to 100%) (Y-axis). The lower chart (abundance) shows the relative abundance of each of the 13 human crAss-like phage genomes (X-axis) in each country (only best-hit reads recruited ≥70% of query coverage were considered). The relative abundances of the human crAss-like phages were normalized by metagenome size (Gbp) and phage length (Kbp).

Determining the presence and abundance of crAss-like phages in each metagenome revealed the most represented crAss-like phage for each country (Fig. 6). However, phage prevalence is not only reflected by relative abundance (Fig. 6, lower chart) but also by the recruitment of the whole genome, which is depicted by black shadowing in the recruitment plots. Plots with only a few black signals indicate that few reads were recruited. Even if the relative abundance bars are high, a phage cannot be assumed to be present in the metagenome. For example, phage cr17 showed a high abundance in USA 1 but was discarded because the reads of this virome were not recruited against the whole phage genome. Given the variations in sequencing depth among the metagenomes (Table 1), the normalized relative abundances are depicted using different Y-axis scales. The metagenome from Spain had the greatest sequencing depth, followed by Finland, Ireland, Mexico, Malawi, and USA 2 (all five being similar), then Uganda and Ethiopia, and USA-1 and Cameroon (both the same). Finally, Tanzania and China metagenomes had the lowest sequencing depths.

Phage cr53 (*Blohavirus americanus,* family *Suoliviridae*) demonstrated high abundance in five countries, including Spain, with high read recruitment, but it was not observed in Malawi, USA 1, Cameroon, Tanzania, and China. Overall, the phage with the highest relative abundance was cr85 (*Kahnovirus oralis,* family *Steigviridae*) (Fig. 5), which is explained by its high prevalence in Spain, but it was below the detection limit in Mexico, Ethiopia, USA-1, Cameroon, Tanzania, and China, and showed low recruitment in Malawi and Ireland. Phages cr6 and cr12 (both of genus *Afonbuvirus*), ranking second in global abundance (Fig. 5), exhibited good levels of recruitment in seven countries, and moderate in three countries. These two phages, which were below the detection limit in only three countries, came the closest to the ideal of universal human-specific crAss-like phage in this study. In summary, whereas certain phages showed high representativity in some countries, they are not recruited in other and there is no human-specific crAss-like phage that has spread to many of the countries or universally.

DNA regions of the prototypical p-crAssphage has been described as having a worldwide distribution [15]. Although p-crAssphage was not included in our study because it was highly recruited in chicken viromes from Spain and China (Supplementary Data 4), we were still interested in exploring the relative abundance of the whole p-crAssphage genome in different metagenomes around the world. In accordance with previous reports [15], the relative abundance of this phage was high in human viromes from Ireland, Spain, and USA 1, but lower in Finland, Malawi, and USA 2 (Supplementary Fig. 4). The entire p-crAssphage genome was below the detection limit in metagenomes from five out of 12 countries (Ethiopia, Cameroon, Tanzania, Mexico, and China).

## Discussion

In 2014, a new virus named crAssphage (p-crAssphage, assigned to the species *Carjivirus communis*) was identified and defined as highly human-specific [1]. Although it has never been isolated, p-crAssphage was the first member of the order Crassvirales to be identified in human feces and the analysis of fragments of its DNA allowed to determine a wide biogeographical distribution [15], particularly in Western countries [13]. Many studies report that the molecular detection of p-crAssphage genome has proven effective in assessing the abundance of this phage in different ecosystems containing human fecal contamination [3, 52–58]. However, there has been a misconception of what is being detected when referring to crAssphage. Many authors refer to p-crAssphage as a single phage, without considering the diversity of phages within the order Crassvirales. The present study is the first to evaluate which members within the crAss-like group other than p-crAssphage are highly and exclusively present in the human gut and to explore the reasons of this specificity.

p-crAssphage and other members of the Crassvirales are highly abundant and specific to the mammalian gut, particularly in humans [4, 15, 59, 60], but not exclusively. A taxonomical analysis of Metagenome Assembled Genomes (MAGs) of crAss-like phages revealed some environmental (potentially non-human) members within the Epsilon and Zeta groups of the Crassvirales order [6]. Additionally, few previous analyses have already detected p-crAssphage in samples containing exclusively animal feces, albeit at lower concentrations than in human feces or urban sewage [17]. Its presence in animal feces has been attributed to a loss of phage specificity for the human gut and to the use of a qPCR assay targeting a non-human-specific region in the p-crAssphage genome, which might be shared by other crAss-like phages present in animals. This observation prompted us not to use genome fragments in the present study, due to their potential low specificity, and instead to analyze complete genomes of crAss-like phages in human samples, as well as to confirm their presence or absence in animal samples.

For recruitment analysis of the highly human-specific phages we used samples with confirmed presence of human pollution from high (human feces, wastewater, sludge) to low fecal content (environmental and food samples). Pools of each type of sample were generated with various datasets to obtain the necessary sequencing effort for nearly complete sequence coverage to detect specific phage genomes. The sequencing effort of all pools (Supplementary Dataset 2) was similar or above the size recommended [61], with the exception of soil viromes. Despite the small size of soil viromes, they were included in the study because some crAss-like phages were detected in soil virome 1 (cutoff ≥70% of query coverage) and with a relative high abundance (Supplementary Fig. 2).

Of the 91 phage genomes analyzed (67 if only one representative of the 25 crAssBcn phages is included), only 41.8% were highly abundant and diverse (ANIr between 85–100%) in the human gut, and some were either not recruited or found in very low concentrations in all viromes. Within this group of human phages, exclusive specificity for the human gut was even less common (only 5.97% showed absolute specificity and 13.4% were partially specific). It is possible that the viromes analyzed may contain new crAss-like phage sequences not yet described and, consequently, not included in our reference database. This study used only previously reported and confirmed complete crAss-like phage genomes.

Several phages found in the human feces with high human-specificity were not among the phages with the highest recruitment levels in wastewater viromes, even if all were present. Of the 13 phage genomes showing higher recruitment in humans, only four were well represented in wastewater viromes (ɸCrAssBcn3, ΦCrAss001, cr112, and cr85, all belonging to the family *Steigviridae*) (Table 2), and only cr112 and cr85, were classified as highly human-specific. In addition, none of the crAss-like phages analyzed were detected by metagenomics in samples with low fecal content, such as soil or food. Ideally, a phage present in fecal samples is expected to be found in human wastewater, containing a high fecal load, and be recruited even in samples with low levels of fecal contamination. Nevertheless, the use of metagenomics has some limitations. As the aim of this study was to evaluate which phages were the most prevalent in humans, metagenomics was a useful tool, allowing us to analyze whole phage genomes. Metagenomic analysis, however, is not suitable for application in real, complex samples (like wastewater) or in those with a low DNA content (soil or food). The high phage diversity of wastewater, reported to be higher than in feces [61–63], reduces the chances of detecting a single phage genome in a wastewater sample. In wastewater samples, as in those with low fecal inputs, the presence of crAss-like phages could be determined using more sensitive detection techniques, such as qPCR amplification. Compared to PCR-based methods, the limit of detection of metagenomics may be a couple of orders of magnitude higher, or even more, depending on DNA yields and sequencing efforts [64]. Considering this, we preferred not to exclude any of the phages showing low relative abundance in wastewater to avoid underestimation of some of the crAss-like phages analyzed and we continued our study with the 28 phages highly recruited in human fecal samples.

It should be considered that the group of 28 phages present in human samples were selected using samples with human contamination from our own geographical area. Because crAss-like phages show geographical variability, it could not be excluded that the use of human samples from other areas may have included some more candidates to the list with higher relative abundances. Even so, the Spanish metavirome has the highest relative abundance among all the countries studied and showed recruitment against all the phages analyzed. In contrast, the use of soil or food viromes from other areas would not have influenced the results because the fecal load of these samples and the limitation of metagenomics is more determinant to identify new candidates than the geographical origin of these samples.

Various animal viromes in public databases were then assessed for the absence of human-related sequences, using only those with high sequence quality (Supplementary Data 2). The animal viromes (from cows, pigs, rabbits, or non-human primates) were largely without crAss-like phages, indicating the absence of human fecal pollution and supporting the preferential residence of Crassvirales in the human gut. Conversely, various crAss-like phages were detected in two chicken viromes, from Spain and China. In these cases, the absence of human fecal contamination cannot be guaranteed, particularly for the samples not analyzed in our laboratory. This finding, together with previous reports of Crassvirales detection in animal samples [16, 17, 54, 65], highlights the need for more research to assess which members of this heterogeneous order are the most exclusive inhabitants of the human gut.

Among different animals, the presence of p-crAssphage in cats could be attributed to contamination from their human companions [16], that transferred the phage or the host strain *B. intestinalis* [16]. Cross-contamination from humans is not that evident in other comparative studies that have also detected high number of crAss-like phages in chicken viromes [17, 65]. In the present work, considering the detection limit of metagenomics, the presence and relative abundance of crAss-like phages in chicken viromes was not negligible (Fig. 2). Overestimation due to cross-reactivity of the qPCR assay can be ruled out, as the whole phage genome was used rather than single DNA fragments. Consequently, in addition to the human gut, the chicken gut could be a reservoir of some crAss-like phages. This possibility is not unexpected, considering the ability of diverse *Bacteroides* species isolated from chickens to adapt to humans and vice-versa [66]. Once the bacterial host is in a new gut environment, the phages infecting this host should have a higher chance of adaptation. However, the phages showed varying prevalence in the two chicken viromes. Thus, if only one of the viromes had been studied, some of the phages would have appeared as highly human-specific. As we did not want to exclude any of the human-specific phages, and we could not verify whether any of the viromes (European or Chinese) were free of human fecal contamination, phages not recruited in one chicken virome were considered to have partial specificity for humans (Fig. 2).

To identify those crAss-like phages most represented in humans, phages that had a minimum of 40% genome coverage by metagenomic reads were considered. Similarly, in animal viromes, phage genomes with less than 40% coverage were regarded as not recruited. This cutoff recruitment value is more stringent than the previously reported 10% coverage by metagenomic reads [67]. Considering that crAss-like phages have a genome size close to 100 kb [5, 59], a single gene could comprise 10% of the genome of a crAss-like phage. This single gene may be shared among several crAss-like phages and be considered within a cutoff of 10% coverage, without being a real phage. We did not want to base our conclusions on a small fraction of the genome that could potentially confuse the results. We decided to increase the requirements for horizontal genomic coverage to consider when a phage genome is present, thereby reducing the risk of overestimating detection and obtaining false positives. In humans, the average genomic coverage was 47%, and in wastewater ~37%. For these reasons, we deemed it safer to increase to a cutoff of 40% the coverage requirement to determine the specificity of crAss-like phages. Based on these thresholds, only 13 human crAss-like phages from the initial group of 91 phage genomes analyzed were selected, whereas p-crAssphage was excluded because it failed to meet the criteria for human specificity and was highly recruited in both chicken viromes (Supplementary Data 4).

Despite the similarities between DAC15 and DAC17, and between ΦCrAssBcn3 and ΦCrAss001, as previously reported [5, 9], a comparison of the 13 human-specific crAss-like phages failed to uncover a shared “core” genome at the nucleotide level that could be correlated to their specificity for the human host. The percentage of identity among the 13 crAss-like phages was minimal, their genomes showing low synteny and very low homology. Moreover, the heterogeneity persisted when comparing only the four crAss-like phages highly specific for humans. This diversity is supported by the phylogenomic analysis of the 13 human phages, that belong to different genera or families of the Crassvirales order. These results suggest that within the Crassvirales group, human specificity is likely not a feature of a common human-resident ancestor before spreading across the globe, but rather was introduced on separate/independent occasions in evolutionary history. The phages belong to different genera and species, and are distributed in three different families (*Steigviridae*, *Intestiviridae*, and *Suoliviridae*), that have evolved independently within the order, possibly achieving human specificity through symbiotic events with their bacterial host strain within the human reservoir. This symbiotic relationship is not limited to a single bacterial species, because in the group of 13 human crAss-like phages at least three different *Bacteroides* species can be identified as hosts (*B. intestinalis, B. tethaiotaomicron*, and *B. xylanisolvens*) [7, 9, 10].

Some crAss-like phage genomes, mostly from the Zeta group, use in some parts of their genome alternative genetic codes, in which stop codons such as TGA or TAG are reassigned and encode an amino acid (tryptophan or glutamine respectively) [6, 68]. In addition to alternative genetic codes, these genomes include massively introns and inteins that seriously complicates annotation [6]. These changes in the genetic code may affect the proteins encoded and should be considered when cataloging phage gene inventories or when analyzing the biological function of the proteins. In our study, we did not annotate phages genomes, but we used the annotation of all crAss-like phage genomes available in the databases, which may have not considered putative read-through stop codons and other elements. For this reason, phage genome comparison was performed at nucleotide level (Fig. 3). The phylogenomic analysis presented in the viral proteomic tree (Fig. 4) were coherent with the comparison performed at nucleotide level.

Few studies have evaluated the spread of Crassvirales around the globe [4, 15]. Our analysis of 1260 human gut metagenomes from 14 countries detected crAss-like phages in metagenomes of 12 countries, with the phages showing varying levels of global distribution. The identification of human-specific crAss-like phages in metagenomes of different countries indicates that the members of the large and diverse Crassvirales group have spread differently across the globe [4, 6, 59], but none is globally distributed. In our study, some countries appear to have higher relative abundances of crAss-like phages than others. The depth of the available viromes influenced the relative abundance of the phages. The metagenome of Spain had the highest relative abundance, followed by Finland, Ireland, Mexico, Malawi, and the USA-2. The relative abundances were normalized by the sequencing depth of the metagenome, the dataset size, and the genome length, but even after normalization, the greater depth influenced the observed relative abundances.

When present, p-crAssphage showed very high relative abundances, higher than any of the other crAss-like phages studied. However, the whole p-crAssphage genome failed to be recruited or fell below the detection threshold in numerous countries analyzed. p-crAssphage has been proposed and extensively used as a universal marker of human fecal pollution. Recently, crAssBcn phages were also found to be highly abundant in Spain and other countries [5]. However, some of the crAss-like phages identified in the present study have a wider geographical distribution and are more represented in human gut viromes than p-crAssphage or than crAssBcn phages. The variability in the geographical distribution of p-crAssphage has been attributed to factors such as human migration patterns, higher fitness, or environmental stability [15].

The Crassvirales group show high levels of bacterial host specialization [7]. Therefore, it can be assumed that the specificity of some crAss-like phages for the human gut is the result of their ability to infect human-specific *Bacteroides* strains, rather than an attribute of the phages themselves. The specificity between the *Bacteroides* group and their hosts [69] enables the isolation of phages specifically present in humans or animals by using a human-specific or an animal-specific *Bacteroides* host strains [70, 71]. The presence of *B. intestinalis* in cats [16] or *Bacteroides* spp. in chicken [66] could explain the presence of crAss-like phages in these animals.

Human-specific *Bacteroides* hosts vary in their sensitivity to different phages according to the geographic area where they have been isolated, being more susceptible to those phages from the same geographic area and of the same fecal origin [70, 72]. Because *Bacteroides* shows a strong adaptation to the gut environment [73], these geographical differences in *Bacteroides* can be attributed to various epigenetic factors affecting their human hosts, such as age, health state, and cultural traditions affecting diet. Diet modulates the gut microbiota profiles of different human populations and promote diverse symbiotic relationships between human hosts and their microorganisms [74, 75].

The emergence and dissemination of human symbiotic *Bacteroides* spp. strains may have occurred within human populations inhabiting distinct geographical areas. As a result, human-specific crAss-like phages infecting these strains would have soon emerged. These events might have occurred stochastically and simultaneously in various human populations occupying diverse ecological niches worldwide, thus contributing to the varied geographical distribution of the different human-specific crAss-like phages and the absence of a phylogenetic relationship between them, as observed in this study.

To properly study crAss-like phages and their biological cycle, it is necessary to use isolated virions. Using an isolated phage is desirable, firstly because its genome is not a product of incorrect assembly, and secondly, its ecological distribution and persistence, host-phage relationship, and biological cycle can be assessed. Thus, the isolated phage can be used as a control in experiments to evaluate analytical performance, and their persistence under different treatments [76, 77]. In the group of human crAss-like phages, ΦCrAssBcn3 (*Kehishuvirus* sp.), ΦCrAss001 (*Kehishuvirus primarius*), ΦCrAss002 (*Jahgtovirus secundus*), DAC15, and DAC17 (both *Wulfhauvirus bangladeshii*) are isolated phages with different *Bacteroides* strains as hosts [5, 10]. Among them, DAC15 and DAC17 were the most human-specific, but the least abundant globally. ΦCrAss002 was the most recruited overall but it was not detected in all the countries, and although it was quite specific for humans, it was also found in chicken.

Soon after its discovery, p-crAssphage was proposed as a marker of human fecal contamination. Tracing the origin of fecal pollution (named “Microbial Source Tracking” or MST [14, 72]) is particularly useful for the rapid prediction of health risks associated with exposure to fecal pollution [78], and consequently the biotechnological application of this group of phages is of pivotal relevance. p-crAssphage and other crAss-like phages have been recognized as useful markers to distinguish between human and animal fecal samples [17, 54, 55, 79]. However, our results raise questions about the human specificity and global distribution of p-crAssphage and consequently its use as a universal human marker, particularly if applied without considering its limitations, and advocate for the investigation of alternative candidates. Regrettably, finding a universal, highly human-specific crAss-like phage seems incompatible with the characteristics of this group of phages. In this scenario, care should be taken to avoid amassing data on crAssphage without assessing human specificity in different regions. Otherwise, there is a risk of selecting an unsuitable marker lacking correlation with human fecal microorganisms, ultimately undermining the potential biological applications of this novel human-specific indicator.

After analyzing 91 crAss-like phage genomes, our aim was to identify those found in the human gut, ideally absent in animal feces, and to elucidate whether common traits of the phages are determinants for their human specificity. At this juncture, this work provides a selection of human crAss-like phages and their geographical distribution. The vast majority of the crAss-like phages in the original list were discarded, leaving a small group of unrelated crAss-like phages, confirming that, unlike previously thought, most phages within Crassvirales are not exclusively found in human viromes.

Our conclusions apply to the group of phages analyzed in this study, and if new crAss-like phage genomes become available in the future, they could lead to different outcomes. Searching for new crAss-like phages in the analyzed viromes could help retrieving sequences of new divergent members of the Crassvirales order [80]. However, identification of new crAss-like phages was not the goal of this study, but to determine the human specificity of the ones already described. In addition, it should be considered the difficulty in reconstructing complete genomes of viruses, such as crAss-like phages, from viromes, as the genomic diversity present in this group of viruses complicates their recovery through metagenomic assembly [81]. It has been reported that analysis of bulk metagenomes may provide an advantage for viral detection in comparison to Virus-like particle (VLP)-enriched metagenomes [80]. However, the abundance of cellular sequences in a bulk metagenome may hinder the detection of viral sequences. In our analysis, most bulk metagenomes did not show recruitment against the selected phages (Table 1), whereas hits were obtained using VLP-enriched metagenomes.

With the group of phages analyzed here, our results suggest that the few human crAss-like phages do not derive from a common human-specific ancestor, but they appear in different lineages within the Crassvirales group. In addition, they do not share common genetic traits that could be attributed to their specific residence in the human gut. The lack of common genetic sequences between the 13 human phages difficults the design of molecular tools for their detection, which would be very useful for their biotechnological application as human fecal marker. There is not a uniform global distribution of the human crAss-like phages either, although some of them are more globally widespread than others and they seem to have emerged simultaneously in different geographical areas. Our results indicate that the presence of human-specific crAss-like phages is possibly depending on the residence of their bacterial host strain, *Bacteroides*, that shows a close symbiotic relationship with the different human populations.

## Supplementary Material

Supplementary_Data_1_rev_wrae192

Supplementary_Data_2_rev_wrae192

Supplementary_Data_3_rev_wrae192

Supplementary_Data_4_rev_wrae192

Supplementary_Figures_revised_wrae192

## Data Availability

All data generated or analyzed during this study are included in this published article and its supplementary information files.

## References

[1] Dutilh BE, Cassman N, McNair K et al. A highly abundant bacteriophage discovered in the unknown sequences of human faecal metagenomes. *Nat Commun* 2014;5:4498. 10.1038/ncomms5498 25058116 PMC4111155

[2] Stachler E, Akyon B, De Carvalho NA et al. Correlation of crAssphage qPCR markers with culturable and molecular indicators of human fecal pollution in an impacted urban watershed. *Environ Sci Technol* 2018;52:7505–12. 10.1021/acs.est.8b00638 29874457

[3] Sabar MA, Honda R, Haramoto E. CrAssphage as an indicator of human-fecal contamination in water environment and virus reduction in wastewater treatment. *Water Res* 2022;221:118827. 10.1016/j.watres.2022.118827 35820313

[4] Guerin E, Shkoporov A, Stockdale SR et al. Biology and taxonomy of crAss-like bacteriophages, the most abundant virus in the human gut. *Cell Host Microbe* 2018;24:653–664.e6. 10.1016/j.chom.2018.10.002 30449316

[5] Ramos-Barbero MD, Gómez-Gómez C, Sala-Comorera L et al. Characterization of crAss-like phage isolates highlights Crassvirales genetic heterogeneity and worldwide distribution. *Nat Commun* 2023;14:4295. 10.1038/s41467-023-40098-z 37463935 PMC10354031

[6] Yutin N, Benler S, Shmakov SA et al. Analysis of metagenome-assembled viral genomes from the human gut reveals diverse putative CrAss-like phages with unique genomic features. *Nat Commun* 2021;12:1044. 10.1038/s41467-021-21350-w 33594055 PMC7886860

[7] Shkoporov AN, Khokhlova EV, Fitzgerald CB et al. ΦCrAss001 represents the most abundant bacteriophage family in the human gut and infects *Bacteroides intestinalis*. *Nat Commun* 2018;9:4781. 10.1038/s41467-018-07225-7 30429469 PMC6235969

[8] Turner D, Shkoporov AN, Lood C et al. Abolishment of morphology-based taxa and change to binomial species names: 2022 taxonomy update of the ICTV bacterial viruses subcommittee. *Arch Virol* 2023;168:74. 10.1007/s00705-022-05694-2 36683075 PMC9868039

[9] Hryckowian AJ, Merrill BD, Porter NT et al. *Bacteroides thetaiotaomicron*-infecting bacteriophage isolates inform sequence-based host range predictions. *Cell Host Microbe* 2020;28:371–379.e5. 10.1016/j.chom.2020.06.011 32652063 PMC8045012

[10] Guerin E, Shkoporov AN, Stockdale SR et al. Isolation and characterisation of ΦcrAss002, a crAss-like phage from the human gut that infects *Bacteroides xylanisolvens*. *Microbiome* 2021;9:89. 10.1186/s40168-021-01036-7 33845877 PMC8042965

[11] Papudeshi B, Vega AA, Souza C et al. Host interactions of novel Crassvirales species belonging to multiple families infecting bacterial host, *Bacteroides cellulosilyticus* WH2. *Microb Genom* 2023;9:001100. 10.1099/mgen.0.001100 37665209 PMC10569736

[12] Holmfeldt K, Solonenko N, Shah M et al. Twelve previously unknown phage genera are ubiquitous in global oceans. *Proc Natl Acad Sci USA* 2013;110:12798–803. 10.1073/pnas.1305956110 23858439 PMC3732932

[13] Smith L, Goldobina E, Govi B et al. Bacteriophages of the order Crassvirales: what do we currently know about this keystone component of the human gut virome? *Biomol Ther* 2023;13:584. 10.3390/biom13040584 PMC10136315 37189332

[14] Stachler E, Bibby K. Metagenomic evaluation of the highly abundant human gut bacteriophage CrAssphage for source tracking of human fecal pollution. *Environ Sci Technol Lett* 2014;1:405–9. 10.1021/ez500266s

[15] Edwards RA, Vega AA, Norman HM et al. Global phylogeography and ancient evolution of the widespread human gut virus crAssphage. *Nat Microbiol* 2019;4:1727–36. 10.1038/s41564-019-0494-6 31285584 PMC7440971

[16] Li Y, Gordon E, Shean RC et al. CrAssphage and its bacterial host in cat feces. *Sci Rep* 2021;11:815. 10.1038/s41598-020-80076-9 33436756 PMC7804022

[17] García-Aljaro C, Ballesté E, Muniesa M et al. Determination of crAssphage in water samples and applicability for tracking human faecal pollution. *Microb Biotechnol* 2017;10:1775–80. 10.1111/1751-7915.12841 28925595 PMC5658656

[18] Blanco-Picazo P, Gómez-Gómez C, Tormo M et al. Prevalence of bacterial genes in the phage fraction of food viromes. *Food Res Int* 2022;156:111342. 10.1016/j.foodres.2022.111342 35651089

[19] Blanco-Picazo P, Fernández-Orth D, Brown-Jaque M et al. Unravelling the consequences of the bacteriophages in human samples. *Sci Rep* 2020;10:6737. 10.1038/s41598-020-63432-7 32317653 PMC7174282

[20] Fernández-Orth D, Miró E, Brown-Jaque M et al. Faecal phageome of healthy individuals: presence of antibiotic resistance genes and variations caused by ciprofloxacin treatment. *J Antimicrob Chemother* 2019;74:854–64. 10.1093/jac/dky540 30649322

[21] Blanco-Picazo P, Gómez-Gómez C, Aguiló-Castillo S et al. Chicken liver is a potential reservoir of bacteriophages and phage-derived particles containing antibiotic resistance genes. *Microb Biotechnol* 2022;15:2464–75. 10.1111/1751-7915.14056 35485188 PMC9437878

[22] Lasobras J, Dellunde J, Jofre J et al. Occurrence and levels of phages proposed as surrogate indicators of enteric viruses in different types of sludges. *J Appl Microbiol* 1999;86:723–9. 10.1046/j.1365-2672.1999.00722.x 10212418

[23] Jofre J. Indicators of waterborne enteric viruses. In: Bosch A (ed). Human Viruses in Water. Amsterdam, Netherlands: Elsevier, 2007, pp. 227–49. 10.1016/S0168-7069(07)17011-7.

[24] Anonymous . ISO 10705-2: Water Quality. Detection and Enumeration of Bacteriophages -Part 2: Enumeration of Somatic Coliphages. Geneva: International Organisation for Standardisation, 2000.

[25] Sambrook J, Russell D. Molecular Cloning: A Laboratory Manual. Cold Spring Harbor, NY: Cold Spring Harbor Laboratory Press, 2001, 999.

[26] Bolger AM, Lohse M, Usadel B. Trimmomatic: a flexible trimmer for Illumina sequence data. *Bioinformatics* 2014;30:2114–20. 10.1093/bioinformatics/btu170 24695404 PMC4103590

[27] Wingett S, Andrews A. FastQ screen: a tool for multi-genome mapping and quality control. *F1000Res* 2018;7:1338. 10.12688/f1000research.15931.1 30254741 PMC6124377

[28] Moraru C, Varsani A, Kropinski AM. VIRIDIC-A novel tool to calculate the intergenomic similarities of prokaryote-infecting viruses. *Viruses* 2020;12:1268. 10.3390/v12111268 33172115 PMC7694805

[29] Rodriguez-R LM, Konstantinidis KT. The enveomics collection: a toolbox for specialized analyses of microbial genomes and metagenomes. *PeerJ Prepr* 2016;4:e1900v1.

[30] Plotly – Collaborative Data Science – 2015 Data Storytelling Studio @ MIT . https://datastudio2015.datatherapy.org/index.html%3Fp=745.html. Accessed 22 Mar 2022.

[31] Meziti A, Tsementzi D, Rodriguez-R LM et al. Quantifying the changes in genetic diversity within sequence-discrete bacterial populations across a spatial and temporal riverine gradient. *ISME J* 2019;13:767–79. 10.1038/s41396-018-0307-6 30397261 PMC6461791

[32] Babicki S, Arndt D, Marcu A et al. Heatmapper: web-enabled heat mapping for all. *Nucleic Acids Res* 2016;44:W147–53. 10.1093/nar/gkw419 27190236 PMC4987948

[33] Li W, Godzik A. Cd-hit: a fast program for clustering and comparing large sets of protein or nucleotide sequences. *Bioinformatics* 2006;22:1658–9. 10.1093/bioinformatics/btl158 16731699

[34] Adriaenssens EM, Rodney BJ. How to name and classify your phage: an informal guide. *Viruses* 2017;9:70. 10.3390/v9040070 28368359 PMC5408676

[35] Darling AE, Mau B, Perna NT. progressiveMauve: multiple genome alignment with gene gain, loss and rearrangement. *PLoS One* 2010;5:e11147. 10.1371/journal.pone.0011147 20593022 PMC2892488

[36] Aiemjoy K, Altan E, Aragie S et al. Viral species richness and composition in young children with loose or watery stool in Ethiopia. *BMC Infect Dis* 2019;19:53. 10.1186/s12879-019-3674-3 30642268 PMC6332554

[37] Chehoud C, Dryga A, Hwang Y et al. Transfer of viral communities between human individuals during fecal microbiota transplantation. *MBio* 2016;7:e00322. 10.1128/mBio.00322-16 27025251 PMC4817255

[38] Fernandes MA, Verstraete SG, Phan T et al. Enteric virome and bacterial microbiota in children with ulcerative colitis and Crohn disease. *J Pediatr Gastroenterol Nutr* 2019;68:30–6. 10.1097/MPG.0000000000002140 30169455 PMC6310095

[39] Han M, Yang P, Zhong C et al. The human gut virome in hypertension. *Front Microbiol* 2018;9:3150. 10.3389/fmicb.2018.03150 30619215 PMC6305721

[40] Kramná L, Kolářová K, Oikarinen S et al. Gut virome sequencing in children with early islet autoimmunity. *Diabetes Care* 2015;38:930–3. 10.2337/dc14-2490 25678103

[41] McCann A, Ryan FJ, Stockdale SR et al. Viromes of one year old infants reveal the impact of birth mode on microbiome diversity. *PeerJ* 2018;6:e4694. 10.7717/peerj.4694 29761040 PMC5944432

[42] Monaco CL, Gootenberg DB, Zhao G et al. Altered virome and bacterial microbiome in human immunodeficiency virus-associated acquired immunodeficiency syndrome. *Cell Host Microbe* 2016;19:311–22. 10.1016/j.chom.2016.02.011 26962942 PMC4821831

[43] Pérez-Brocal V, García-López R, Vázquez-Castellanos JF et al. Study of the viral and microbial communities associated with Crohn’s disease: a metagenomic approach. *Clin Transl Gastroenterol* 2013;4:e36. 10.1038/ctg.2013.9 23760301 PMC3696940

[44] Rampelli S, Turroni S, Schnorr SL et al. Characterization of the human DNA gut virome across populations with different subsistence strategies and geographical origin. *Environ Microbiol* 2017;19:4728–35. 10.1111/1462-2920.13938 28967228

[45] Reyes A, Blanton LV, Cao S et al. Gut DNA viromes of Malawian twins discordant for severe acute malnutrition. *Proc Natl Acad Sci USA* 2015;112:11941–6. 10.1073/pnas.1514285112 26351661 PMC4586842

[46] Shkoporov AN, Ryan FJ, Draper LA et al. Reproducible protocols for metagenomic analysis of human faecal phageomes. *Microbiome* 2018;6:68. 10.1186/s40168-018-0446-z 29631623 PMC5892011

[47] Yinda CK, Vanhulle E, Conceição-Neto N et al. Gut Virome analysis of Cameroonians reveals high diversity of enteric viruses, including potential interspecies transmitted viruses. *mSphere* 2019;4:e00585. 10.1128/mSphere.00585-18 30674646 PMC6344602

[48] Zhao G, Vatanen T, Droit L et al. Intestinal virome changes precede autoimmunity in type I diabetes-susceptible children. *Proc Natl Acad Sci USA* 2017;114:E6166–75. 10.1073/pnas.1706359114 28696303 PMC5544325

[49] Bikel S, López-Leal G, Cornejo-Granados F et al. Gut dsDNA virome shows diversity and richness alterations associated with childhood obesity and metabolic syndrome. *iScience* 2021;24:102900. 10.1016/j.isci.2021.102900 34409269 PMC8361208

[50] Blanco-Picazo P, Morales-Cortes S, Ramos-Barbero MD et al. Dominance of phage particles carrying antibiotic resistance genes in the viromes of retail food sources. *ISME J* 2023;17:195–203. 10.1038/s41396-022-01338-0 36289309 PMC9860054

[51] Lefkowitz EJ, Dempsey DM, Hendrickson RC et al. Virus taxonomy: the database of the international committee on taxonomy of viruses (ICTV). *Nucleic Acids Res* 2018;46:D708–17. 10.1093/nar/gkx932 29040670 PMC5753373

[52] Ballesté E, Pascual-Benito M, Martín-Díaz J et al. Dynamics of crAssphage as a human source tracking marker in potentially faecally polluted environments. *Water Res* 2019;155:233–44. 10.1016/j.watres.2019.02.042 30851594

[53] Park GW, Ng TFF, Freeland AL et al. CrAssphage as a novel tool to detect human fecal contamination on environmental surfaces and hands. *Emerg Infect Dis* 2020;26:1731–9. 10.3201/eid2608.200346 32511090 PMC7392416

[54] Ahmed W, Payyappat S, Cassidy M et al. Novel crAssphage marker genes ascertain sewage pollution in a recreational lake receiving urban stormwater runoff. *Water Res* 2018;145:769–78. 10.1016/j.watres.2018.08.049 30223182

[55] Sala-Comorera L, Reynolds LJ, Martin NA et al. crAssphage as a human molecular marker to evaluate temporal and spatial variability in faecal contamination of urban marine bathing waters. *Sci Total Environ* 2021;789:147828. 10.1016/j.scitotenv.2021.147828 34052479

[56] Martin NA, Sala-Comorera L, Gao G et al. Inclusion of hydrodynamic properties of bathing waters is critical in selecting faecal indicators to assess public health impacts of faecal contamination. *Water Res* 2023;242:120137. 10.1016/j.watres.2023.120137 37300999

[57] Meuchi Y, Nakada M, Kuroda K et al. Applicability of F-specific bacteriophage subgroups, PMMoV and crAssphage as indicators of source specific fecal contamination and viral inactivation in rivers in Japan. *PLoS One* 2023;18:e0288454. 10.1371/journal.pone.0288454 37450468 PMC10348522

[58] Chettleburgh C, Ma SX, Swinwood-Sky M et al. Evaluation of four human-associated fecal biomarkers in wastewater in southern Ontario. *Sci Total Environ* 2023;904:166542. 10.1016/j.scitotenv.2023.166542 37660819

[59] Koonin EV, Yutin N. The crAss-like phage group: how metagenomics reshaped the human virome. *Trends Microbiol* 2020;28:349–59. 10.1016/j.tim.2020.01.010 32298613

[60] Yutin N, Makarova KS, Gussow AB et al. Discovery of an expansive bacteriophage family that includes the most abundant viruses from the human gut. *Nat Microbiol* 2018;3:38–46. 10.1038/s41564-017-0053-y 29133882 PMC5736458

[61] Rodriguez-R LM, Konstantinidis KT. Nonpareil: a redundancy-based approach to assess the level of coverage in metagenomic datasets. *Bioinformatics* 2014;30:629–35. 10.1093/bioinformatics/btt584 24123672

[62] Newton RJ, McLellan SL, Dila DK et al. Sewage reflects the microbiomes of human populations. *MBio* 2015;6:e02574–14. 10.1128/mBio.02574-14 25714718 PMC4358014

[63] Zaheer R, Lakin SM, Polo RO et al. Comparative diversity of microbiomes and resistomes in beef feedlots, downstream environments and urban sewage influent. *BMC Microbiol* 2019;19:197. 10.1186/s12866-019-1548-x 31455230 PMC6712873

[64] Lindner BG, Gerhardt K, Feistel DJ et al. A user’s guide to the bioinformatic analysis of shotgun metagenomic sequence data for bacterial pathogen detection. *Int J Food Microbiol* 2024;410:110488. 10.1016/j.ijfoodmicro.2023.110488 38035404

[65] Ahmed W, Lobos A, Senkbeil J et al. Evaluation of the novel crAssphage marker for sewage pollution tracking in storm drain outfalls in Tampa. *Florida Water Res* 2018;131:142–50. 10.1016/j.watres.2017.12.011 29281808

[66] Kollarcikova M, Faldynova M, Matiasovicova J et al. Different *Bacteroides* species colonise human and chicken intestinal tract. *Microorganisms* 2020;8:1483. 10.3390/microorganisms8101483 32992519 PMC7600693

[67] Castro JC, Rodriguez LM, Harvey WT et al. imGLAD: accurate detection and quantification of target organisms in metagenomes. *PeerJ* 2018;6:e5882. 10.7717/peerj.5882 30405973 PMC6216955

[68] Peters SL, Borges AL, Giannone RJ et al. Experimental validation that human microbiome phages use alternative genetic coding. *Nat Commun* 2022;13:5710. 10.1038/s41467-022-32979-6 36175428 PMC9523058

[69] Xu J, Bjursell MK, Himrod J et al. A genomic view of the human-*Bacteroides thetaiotaomicron* symbiosis. *Science* 1979;299:2074–6. 10.1126/science.1080029 12663928

[70] Payan A, Ebdon J, Taylor H et al. Method for isolation of *Bacteroides* bacteriophage host strains suitable for tracking sources of fecal pollution in water. *Appl Environ Microbiol* 2005;71:5659–62. 10.1128/AEM.71.9.5659-5662.2005 16151173 PMC1214671

[71] Gómez-Doñate M, Payán A, Cortés I et al. Isolation of bacteriophage host strains of *Bacteroides* species suitable for tracking sources of animal faecal pollution in water. *Environ Microbiol* 2011;13:1622–31. 10.1111/j.1462-2920.2011.02474.x 21443742

[72] Jofre J, Blanch AR, Lucena F et al. Bacteriophages infecting *Bacteroides* as a marker for microbial source tracking. *Water Res* 2014;55:1–11. 10.1016/j.watres.2014.02.006 24583570

[73] Wexler AG, Goodman AL. An insider’s perspective: *Bacteroides* as a window into the microbiome. *Nat Microbiol* 2017;2:17026. 10.1038/nmicrobiol.2017.26 28440278 PMC5679392

[74] Wu M, McNulty NP, Rodionov DA et al. Genetic determinants of in vivo fitness and diet responsiveness in multiple human gut *Bacteroides*. *Science* 2015;350:aac5992. 10.1126/science.aac5992 26430127 PMC4608238

[75] Sonnenburg ED, Zheng H, Joglekar P et al. Specificity of polysaccharide use in intestinal *Bacteroides* species determines diet-induced microbiota alterations. *Cell* 2010;141:1241–52. 10.1016/j.cell.2010.05.005 20603004 PMC2900928

[76] Pascual-Benito M, García-Aljaro C, Casanovas-Massana S et al. Effect of hygienization treatment on the recovery and/or regrowth of microbial indicators in sewage sludge. *J Appl Microbiol* 2015;118:412–8. 10.1111/jam.12708 25443658

[77] Sommer R, Lhotsky M, Haider T et al. UV inactivation, liquid-holding recovery, and photoreactivation of *Escherichia coli* O157 and other pathogenic *Escherichia coli* strains in water. *J Food Prot* 2000;63:1015–20. 10.4315/0362-028X-63.8.1015 10945573

[78] Harwood VJ, Staley C, Badgley BD et al. Microbial source tracking markers for detection of fecal contamination in environmental waters: relationships between pathogens and human health outcomes. *FEMS Microbiol Rev* 2014;38:1–40. 10.1111/1574-6976.12031 23815638

[79] Stachler E, Kelty C, Sivaganesan M et al. Quantitative CrAssphage PCR assays for human fecal pollution measurement. *Environ Sci Technol* 2017;51:9146–54. 10.1021/acs.est.7b02703 28700235 PMC7350147

[80] Gregory AC, Zablocki O, Zayed AA et al. The gut virome database reveals age-dependent patterns of virome diversity in the human gut. *Cell Host Microbe* 2020;28:724–740.e8. 10.1016/j.chom.2020.08.003 32841606 PMC7443397

[81] Martinez-Hernandez F, Fornas O, Lluesma Gomez M et al. Single-virus genomics reveals hidden cosmopolitan and abundant viruses. *Nat Commun* 2017;8:15892. 10.1038/ncomms15892 28643787 PMC5490008

