# Supplementary material for: Recruitment of complete crAss-like phage genomes reveals their presence in chicken viromes, few human-specific phages, and lack of universal detection
Source: ISME J. 2024 Oct 3;18(1):wrae192. doi: 10.1093/ismejo/wrae192 (PMC11475920; doi:10.1093/ismejo/wrae192)
Supplement: Supplementary_Figures_revised_wrae192 [file supplementary_figures_revised_wrae192.pdf]

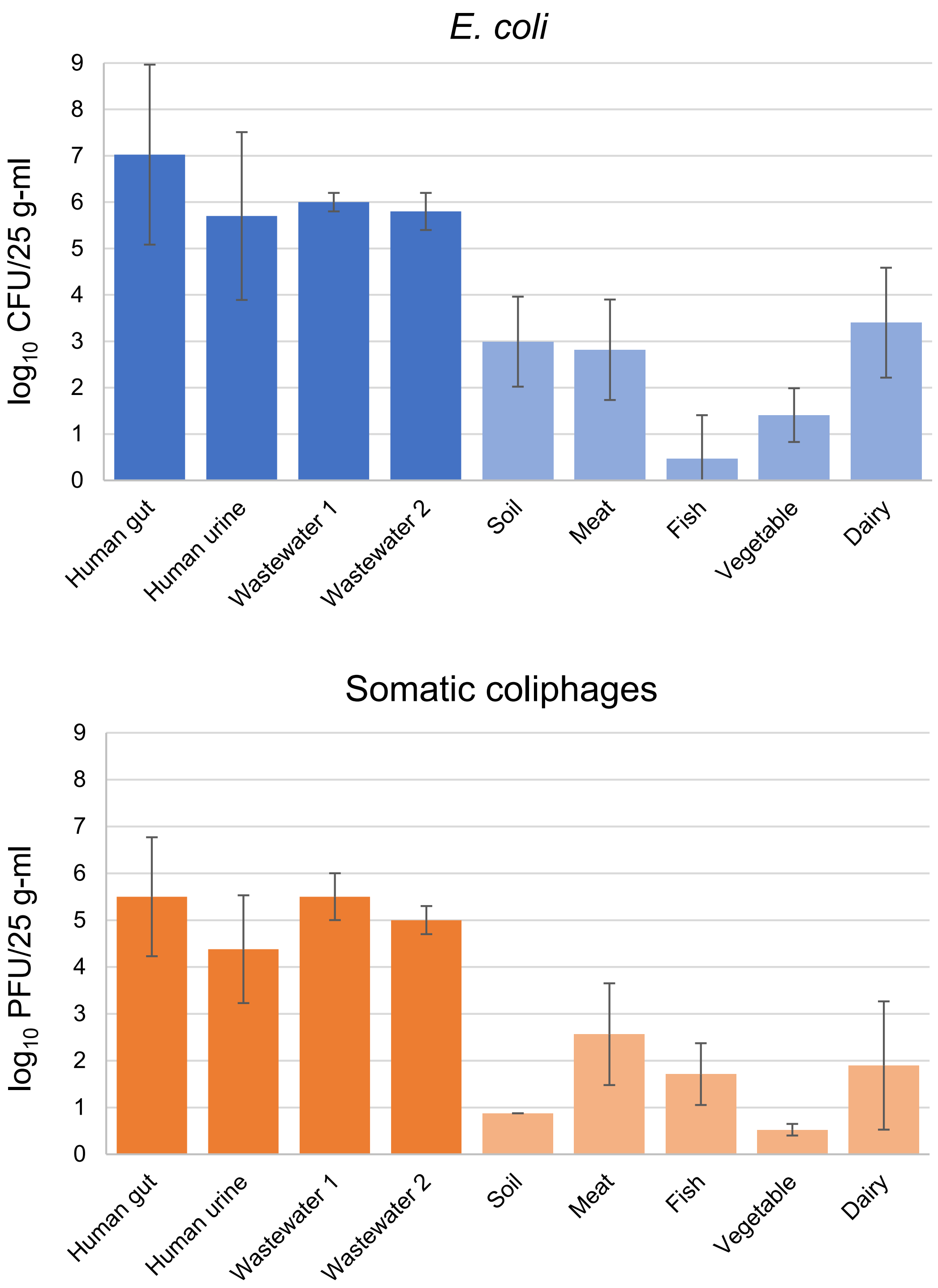

Supplementary Fig. 1. Levels of fecal indicators in the samples used to extract the viromes. *E. coli* (log_{10} CFU/25 g-ml) was used as bacterial fecal indicator while somatic coliphages (log_{10} PFU/25g-ml) were used as viral fecal indicator. Samples were grouped considering their origin.

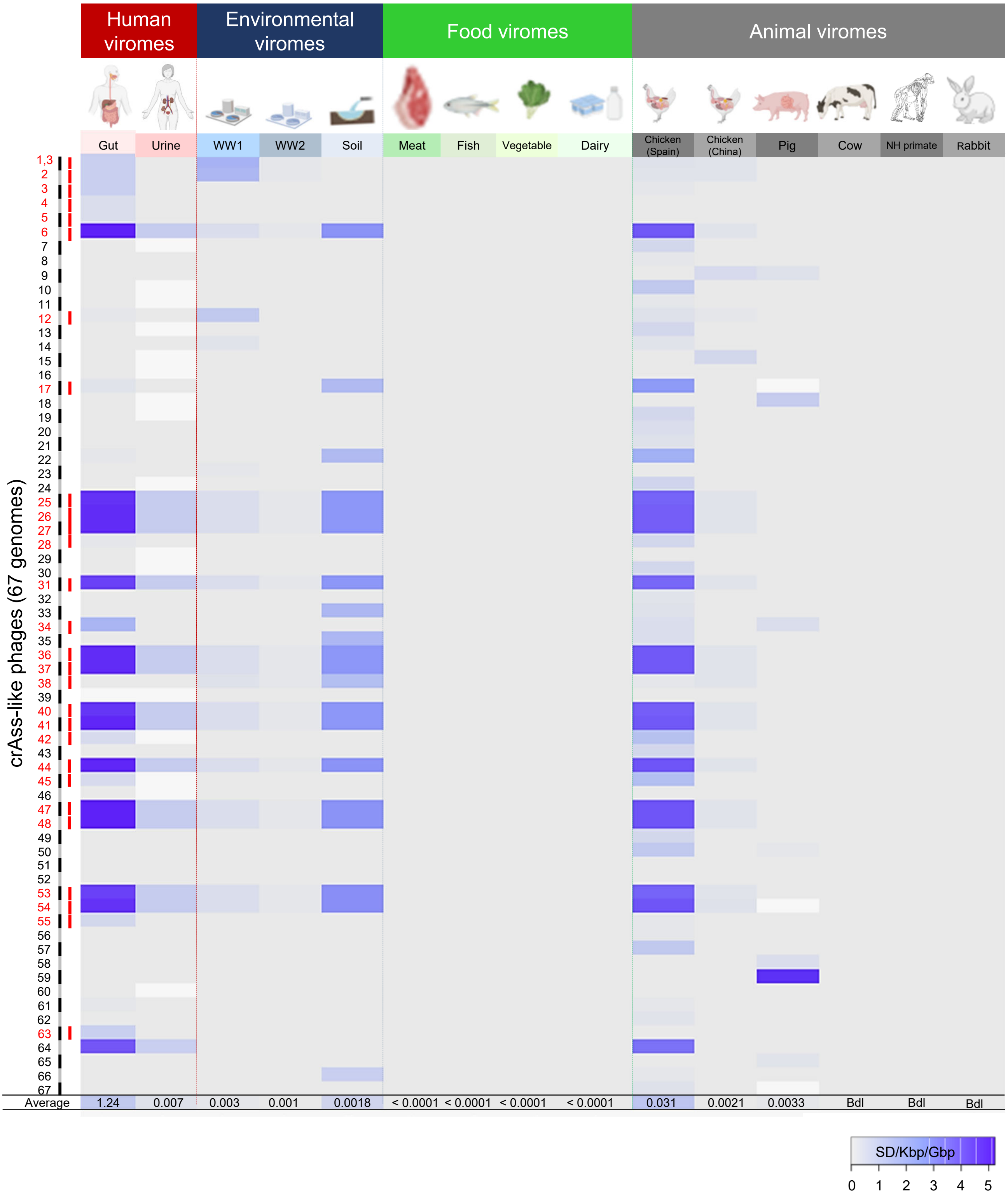

Supplementary Fig. 2. Relative abundance of all crAss-like phages in the viromes. Each line of the chart shows the relative abundance (normalized by the by metagenome size (Gbp) and phage length (Kbp)) of each of the 91 crAss-like genomes from the databases in the different viromes analyzed. Lower row indicates the averaged relative abundance of each virome (SD/Kb/Gbp). Bdl: Below detection limit. WW. Wastewater treatment plant,

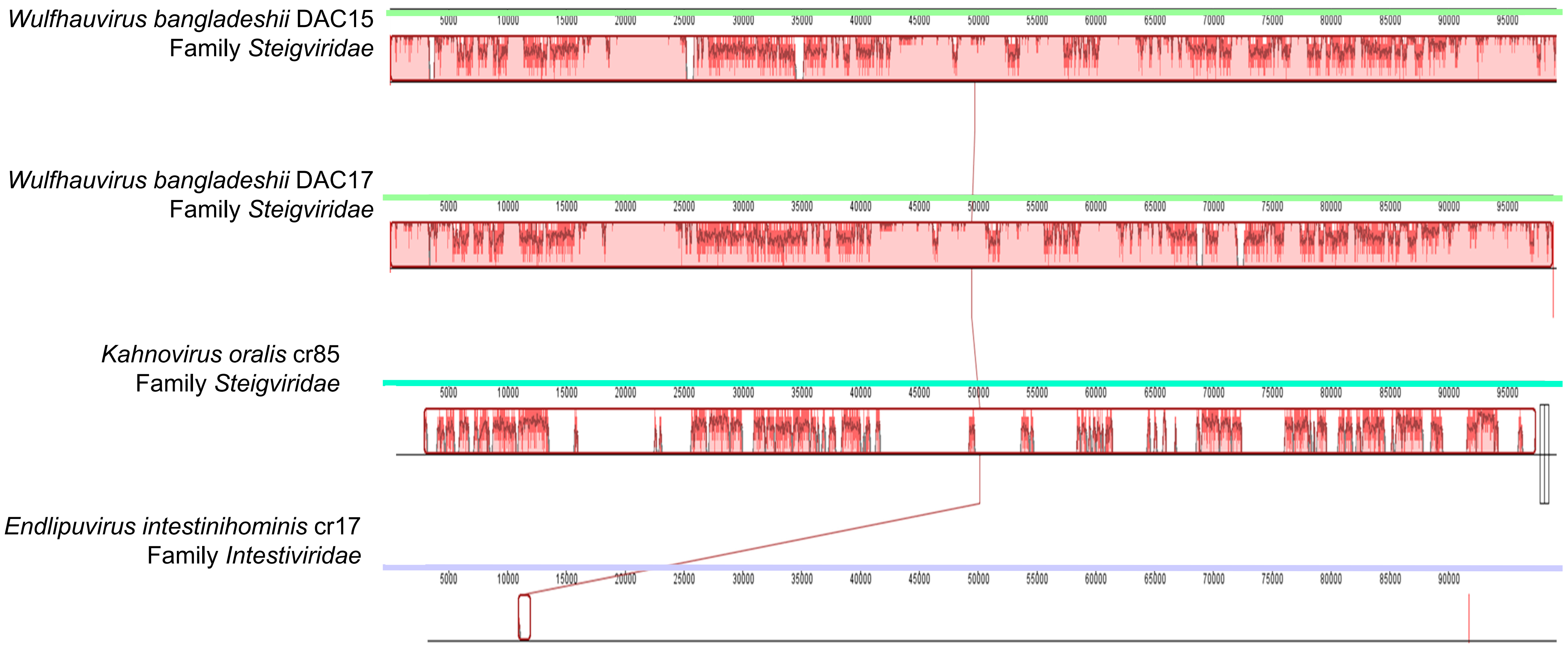

Supplementary Figure 3. Genomic comparison of the genome of the best four selected crAss-like phage. The multialignment of phage genomes were constructed with the complete genomes of the highly human specific crAss-like phages (absent in all animal viromes) by Mauve tool.

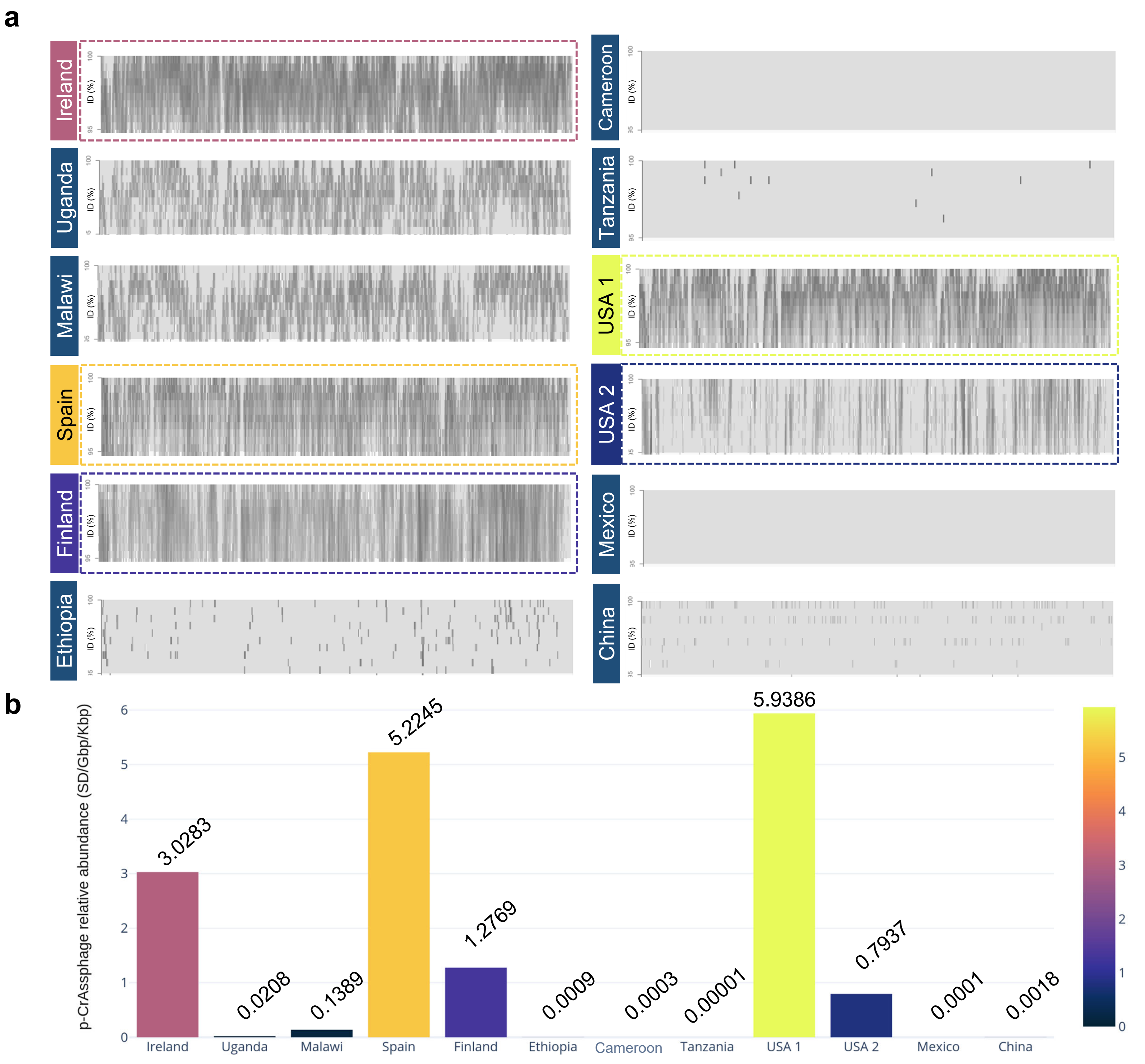

Supplementary Figure 4. **Geographical distribution of the prototypical p-crAssphage around the globe**. **(a)** Fragment recruitment in gut metagenomes from around the globe against the prototypical p-crAssphage **(b)** Relative abundance of the prototypical p-crAssphage in the different countries showing recruitments. Only mapped sequences with coverage of at least 70 % and best-hit reads were considered. Relative abundances of selected crAss-like phages were normalized by metagenome size (Gbp) and phage length (Kbp).
